# Ultrasound-Guided Regional Anesthesia in a Resource-Limited Hospital: Prospective Pilot Study of a Hybrid Training Program

**DOI:** 10.2196/84181

**Published:** 2026-01-08

**Authors:** Jakob E Gamboa, Inge Tamm-Daniels, Roland Flores, Nancy G Sarat Diaz, Mario A Villasenor, Mitchell A Gist, Aidan B Hoie, Christopher Kurinec, Colby G Simmons

**Affiliations:** 1 Department of Anesthesiology University of Colorado School of Medicine Aurora, CO United States; 2 Department of Anesthesiology Hospital Nacional de Coatepeque Coatepeque, Quetzaltenango Guatemala; 3 Department of Biostatistics and Informatics Colorado School of Public Health Aurora, CO United States

**Keywords:** health education, regional anesthesia, resource-limited settings, capacity building, global health

## Abstract

**Background:**

Ultrasound-guided regional anesthesia (UGRA) remains underused in low- and middle-income countries due to barriers to training and equipment. Recent advances in portable ultrasound devices and international partnerships have expanded access to UGRA, enhancing patient safety and quality of care.

**Objective:**

This study describes the development and outcomes of a hybrid UGRA training program for anesthesiologists at the Hospital Nacional de Coatepeque (HNC) in Guatemala.

**Methods:**

An educational pilot program for UGRA was developed based on local needs and feedback, comprising 4 weeks of online modules, an in-person educational conference, and 1 month of supervised clinical practice. Evaluation followed the Kirkpatrick framework using preprogram and postprogram surveys adapted from the Global Regional Anesthesia Curricular Engagement model. Outcomes included participants’ satisfaction, change in knowledge and skill, and procedural performance. Knowledge and skill assessments were compared before and after the training, and clinical data were recorded for 10 months. Nonparametric tests were used to assess changes and associations with performance outcomes.

**Results:**

All 7 anesthesiologists at HNC completed the training program. Knowledge test scores improved by a median percentage increase of 20.8% (IQR 13.5%-28.1%; *r*=0.899; *P*=.02), and procedural skill rating scores increased by a median percentage of 147.1% (IQR 96.9%-197.3%; *r*=0.904; *P*=.03) at 1 month and 131.4% (IQR 90.5%-172.3%; *r*=0.909; *P*=.04) at 4 months after the program. Participants self-reported high satisfaction and substantial clinical improvement and motivation. A total of 54 peripheral nerve blocks were performed under direct supervision in the first month, with 187 blocks recorded over 10 months. The supraclavicular brachial plexus block was the most frequently used (66/187, 35.3%) and replaced the standard general anesthetic for upper extremity surgery in 70 patients. The procedure success rate was 96.3% (180/187), and there were no observed patient complications.

**Conclusions:**

This hybrid curriculum enabled the successful implementation of UGRA at a public hospital in Guatemala, safely expanding clinical capabilities and reducing reliance on general anesthesia for upper extremity surgery. This practical training model provides a framework for implementing UGRA in similar resource-limited hospitals.

## Introduction

Ultrasound-guided regional anesthesia (UGRA) has become the standard of care for safe and effective perioperative pain management in high-income countries. It is frequently used as the primary anesthetic in patients undergoing extremity surgeries [1]. Beyond the operating room, regional anesthesia is increasingly performed by nonanesthesia clinicians, including emergency physicians, to provide timely pain relief in acute care settings [2,3]. Ultrasound-guided peripheral nerve blocks (PNBs) are now used in emergency departments for conditions such as hip and rib fractures and wound management, demonstrating the growing interdisciplinary role of UGRA worldwide [4-6]. However, limited availability of ultrasound equipment and the lack of formal training programs contribute to the underuse of PNBs in low- and middle-income countries (LMICs) [7-10]. The emergence of portable, reliable, and cost-effective ultrasound devices offers new opportunities to expand the use of UGRA in diverse and resource-limited settings [11]. Advancing global regional anesthesia capacity can improve patient care by providing an alternative to general anesthesia, reducing cost and resource consumption, and enhancing patient comfort and safety [1,12].

Addressing the need for training opportunities is critical to the adoption of regional anesthesia techniques in LMICs. International partnerships help improve global capabilities by promoting educational activities and access to supplies. Although several initiatives have attempted to implement UGRA in diverse settings, few have been formally evaluated, and published evidence on their impact in LMICs remains limited [12]. Brouillette et al [13] described the successful development of an in-person educational program called the Global Regional Anesthesia Curricular Engagement (GRACE). In this program, anesthesia providers in Ghana were trained in UGRA techniques, demonstrating positive outcomes and increased procedure volume. Other collaborations have explored fully remote training models delivered via online communication platforms [14,15]. However, to our knowledge, the use of a hybrid training curriculum for UGRA, combining online didactic instruction with in-person, hands-on training, has not been described. Furthermore, most existing reports focus on programs based in African countries at large teaching hospitals with preexisting ultrasound capabilities and delivered in English. There is a paucity of evidence describing educational initiatives developed specifically for medical professionals in Latin America.

Our program was delivered in Guatemala, a Central American country where approximately 90% of the population relies on the public health sector, and approximately half of the country’s 17 million inhabitants live in rural or impoverished communities [16]. Geographic and financial barriers limit access to advanced medical technologies such as ultrasound, which are largely concentrated in metropolitan areas [17]. Additionally, most physician training opportunities are offered in large urban hospitals near the capital of Guatemala City [17]. As a result, smaller public hospitals across the country have limited capacity and access to UGRA. Despite these challenges, there is substantial interest and growing demand for ultrasound-guided procedures nationwide.

In the southwestern region of Guatemala, the Hospital Nacional de Coatepeque (HNC) serves as the regional public hospital, facing high surgical demands amid notable resource constraints. In 2023, a needs assessment was performed through a partnership between the department of anesthesiology at the University of Colorado (CU) and HNC and identified a lack of UGRA capabilities in the region. In response, the department of anesthesiology at the HNC requested a formal training program to build local expertise and improve the quality of perioperative care. Our objective was to develop a tailored hybrid training program to establish a sustainable UGRA service at HNC. This evaluation assessed the outcomes and impact of this pilot program. We hypothesized that the implementation of this hybrid training model would increase clinical knowledge and confidence, enhance skill acquisition, and enable the successful adoption of PNBs in this resource-limited setting in Guatemala.

## Methods

### Overview

We conducted a prospective pilot study of a novel pilot training program for UGRA delivered at HNC in Coatepeque, Guatemala, from April 2024 through June 2024. The evaluation design and materials were adapted from the GRACE model developed by Brouillette et al [13], with the authors’ permission.

### Setting

HNC is the only public surgical hospital in the town of Coatepeque, Quetzaltenango, Guatemala, and serves as the primary referral center for an estimated 150,000 people in this region. A large proportion of patients come from underserved, agriculture-based communities that experience some of the highest rates of poverty and poorest health outcomes in the country [18]. This hospital has 2 main operating rooms and an additional operating room in the obstetric unit for cesarean deliveries. Based on internal hospital records, approximately 5300 surgeries are performed annually across general surgery, obstetrics and gynecology, and orthopedics and traumatology. Trauma and orthopedic extremity procedures represent a major portion of surgical volume due to the high incidence of motor vehicle accidents in this area. Neuraxial anesthesia is used preferentially when feasible, although general anesthesia is typically required for upper extremity surgery. However, due to limitations in supplies, monitoring equipment, and postoperative nursing capacity at this hospital, the use of long-acting opioid analgesics, deep sedation, and general anesthesia is avoided when possible to optimize patient safety [19].

### Needs Assessment

The initial site needs assessment was conducted in February 2023 and identified both strong interest and need for UGRA. A follow-up visit with faculty from CU in August 2023 focused on developing a collaborative action plan, gathering feedback, taking inventory of available supplies, and obtaining approval from hospital leadership. The primary barrier to implementation was the absence of an ultrasound machine due to limited purchasing access. The anesthesia department already maintained a supply of nerve block needles and local anesthetics for infrequent use of nerve stimulation for distal lower extremity blocks. Standard emergency medications were available, except for lipid emulsions. However, no procedure logs or patient consent forms were in place because a regional anesthesia workflow had not yet been established.

To inform curriculum design, an online survey was distributed to HNC anesthesiologists to assess baseline experience and learning preferences (Multimedia Appendix 1). Before training, participants were asked to complete the questionnaire to capture demographic information, self-reported practices, educational needs, and motivations for participation.

### Program Design and Implementation

#### Curriculum Development

**Figure 1:**
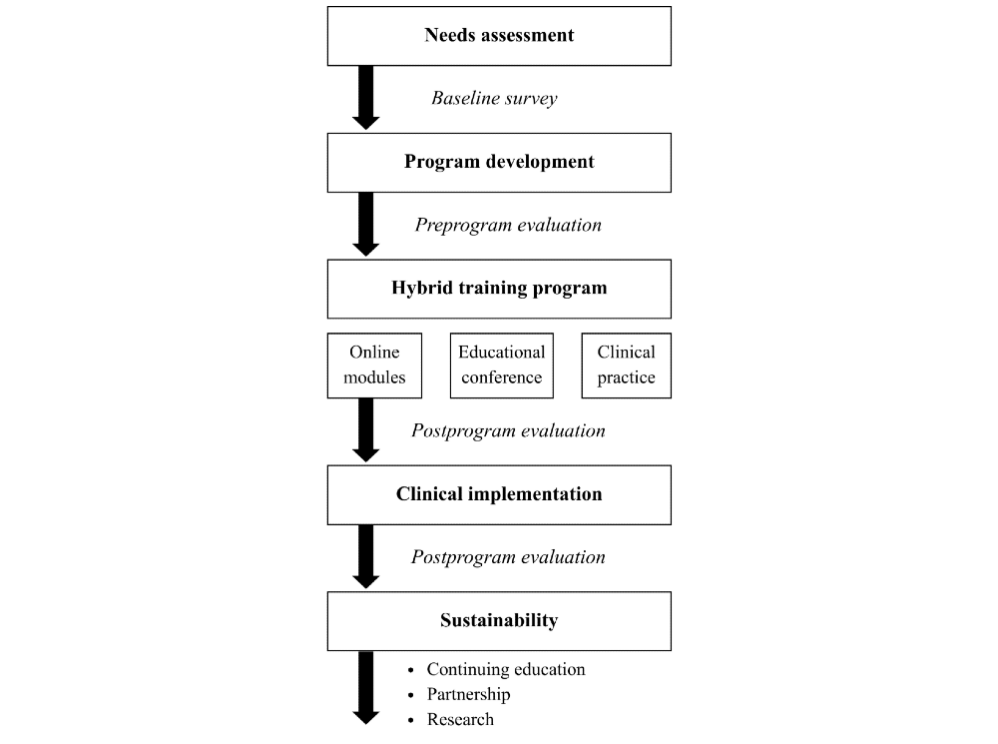
Flowchart of the program design and evaluation.

Information gathered from site assessments, planning meetings, and the online survey was used to develop a hybrid training curriculum. The curriculum was designed collaboratively with local stakeholders and regional anesthesia specialists from CU and subsequently revised based on feedback. All 7 physician anesthesiologists at HNC were invited to participate and provided written consent. The program consisted of three core components: (1) online self-directed learning modules, (2) an in-person educational conference, and (3) clinical practice with direct supervision and bedside teaching (Figure 1). Training was conducted over a 2-month period from April 2024 to May 2024, with a follow-up site visit 4 months after clinical implementation. To simplify the program and emphasize high-yield applications, a limited number of blocks were selected based on the needs assessment and team discussions. These included interscalene and supraclavicular blocks of the brachial plexus, femoral, saphenous (adductor canal), and popliteal blocks.

#### Online Modules

The online learning curriculum was delivered as 4 weekly learning modules on a web-based platform to provide open access to independent learning. As no existing materials met our program objectives for topics or in the Spanish language, we created a dedicated website to address this need [20]. Content for weekly modules was derived from open access resources, such as publications from the New York School of Regional Anesthesia (Multimedia Appendix 2). Additionally, the option for virtual chats or question-and-answer sessions was offered weekly on Fridays at the end of each module.

#### Educational Conference

The educational conference consisted of a 2-day, 16-hour in-person program that included lectures, clinical demonstrations, and hands-on workshops. Sessions were open to anesthesiologists, nurse anesthetists, nursing staff, and surgeons (refer to Multimedia Appendix 3 for conference outline).

#### Clinical Practice

Clinical practice was initiated following the conference, with HNC participants performing nerve blocks during work hours under the supervision of CU preceptors. The program faculty comprised multiple physician anesthesiologists with fellowship training or proficiency in regional anesthesia, ensuring the presence of at least one supervising preceptor during a 4-week period for continuity. Following 1 month of direct supervision, ongoing training and support were offered remotely to address emerging needs. A week-long follow-up visit was completed 4 months after clinical implementation to provide additional support and evaluations.

#### Equipment Donation

To support program initiation, CU faculty donated essential supplies, including a portable ultrasound machine, tablet, nerve block needles, gel, lidocaine, bupivacaine, and lipid emulsion. In response to site visits and internal requests, hospital leadership committed to increasing local supplies and purchased additional nerve block needles and local anesthetics. Additionally, operating room schedules of the surgical services were adjusted to increase time allotted to orthopedic surgery and increase PNB volume during the clinical practice phase.

### Evaluation Framework

#### Evaluation Approach

The evaluation framework and instruments were adapted from the GRACE model, with approval from the study authors [13]. This model used the method proposed by Kirkpatrick and Kirkpatrick [21], a globally recognized and validated approach for evaluating the effectiveness of educational and training programs across disciplines [22]. This model evaluates outcomes across four levels: (1) reaction, (2) learning, (3) behavior, and (4) outcomes.

#### Reaction

Participant reactions were assessed through postprogram satisfaction surveys to solicit perceptions and feedback related to the training. This survey was adapted from the original GRACE satisfaction survey, which contained 10 statements where trainees rate their level of agreement on a 5-point Likert scale and 3 open-ended questions for program feedback and suggestions. Two open-ended questions were also added to describe the most and least helpful components of the training (Multimedia Appendix 4).

#### Learning

Participant learning was evaluated by analyzing changes in knowledge and clinical skills. Knowledge tests were administered before the program and 1 month after clinical implementation of UGRA. The knowledge test consisted of 24 multiple-choice questions that were adapted from the original GRACE instrument. Modifications were made to remove content not taught in this program, provide clarifications, and include items addressing identified knowledge gaps (Multimedia Appendix 5). This test was administered through an online secure platform (Qualtrics XM Platform). Procedural skill acquisition was assessed using a validated global rating scale (GRS) for UGRA developed by Chuan et al [23]. This instrument evaluates 7 domains—preparation, respect for tissue, handling, time and motion, instrument handling, flow, knowledge, and overall performance—graded on a 5-point scale (Multimedia Appendix 6). Participants were observed during procedures and graded at program initiation, 1 month, and 4 months. To further capture participants’ perceptions of the program, additional postsurvey questions were included to self-report changes in confidence, knowledge, and procedural skills using a 5-point Likert scale. All adapted GRACE evaluation instruments were translated into Spanish by 2 language-certified researchers and reviewed for cultural and linguistic accuracy by a local physician coinvestigator.

#### Behavior

Participants were prompted to report their level of interest, plans to continue performing PNBs, and perceived impact on patient care on the postprogram survey at program completion. The number of procedures performed by the anesthesiologist was recorded to assess adoption into practice.

#### Clinical Outcomes

Clinical outcomes were analyzed by total number and type of PNB, success and complication rate, and anesthesia method for 10 months after clinical implementation. Block success was defined a priori by the local department of anesthesiology at the HNC as a patient’s ability to tolerate surgery without general anesthesia or an analgesic block with moderate to complete pain relief. To address the lack of workflow, a block documentation form and clinical log were developed in Spanish based on the New York School of Regional Anesthesia Universal Documentation Sheet for Peripheral Nerve Blocks to record and track procedure information, and patient consent forms were adapted from CU consent documentation (Multimedia Appendices 7 and 8) [24]. Additionally, a safety surveillance system was implemented to evaluate for complications and ensure resolution of the nerve block and lack of new neurological symptoms before discharge. Additional training was provided to nursing staff by a specialized CU postanesthesia care unit nurse to manage and identify complications in patients who received PNBs.

### Statistical Analysis

Descriptive statistics were calculated and reported as frequencies, percentages, and means with SDs. Preprogram and postprogram knowledge test and GRS scores were summarized using medians and ranges. Changes in scores between preprogram and postprogram tests were assessed using paired Wilcoxon signed-rank tests. Likert scale outcomes across multiple time points were evaluated with the Friedman test, followed by pairwise Wilcoxon signed-rank tests for post hoc comparisons. Effect sizes for the Friedman test (Kendall W) and for Wilcoxon signed-rank tests were quantified with 95% CIs estimated using the percentile bootstrap method from 1000 resamples [25,26]. Standardized effect sizes were interpreted using Cohen general guidelines (0.2=weak, 0.5=medium, and 0.8=strong) while recognizing that these thresholds are context dependent and should be interpreted cautiously. Interrater reliability (IRR) for preprogram GRS items, assessed across 2 fixed raters, was quantified using a 2-way mixed-effects intracorrelation coefficient and weighted κ, with 95% CIs obtained using the percentile bootstrap method from 1000 resamples. Two-sided *P* values <.05 were considered statistically significant, with post hoc comparisons corrected for false discovery rate. Given the small sample size, these results may not be generalizable and should be interpreted as exploratory. Statistical analyses were performed using R (version 4.5.1; R Foundation for Statistical Computing). The cohort sample size was determined by the number of anesthesiologists available to perform PNBs at HNC.

### Ethical Considerations

This evaluation received approval from the local research ethics committee (001-2024) and was considered exempt by the Colorado Multiple Institutional Review Board (24-0212). All participants provided written informed consent before enrollment. To protect participant privacy, all data were deidentified and managed in accordance with institutional data security standards, with access restricted to authorized study personnel. Results are reported in aggregate to prevent individual identification. Participants did not receive financial or other compensation for participation in the study.

## Results

### Overview

All anesthesiologists from the HNC completed the training, with a 100% (7/7) response rate for surveys and evaluations. Baseline participant characteristics are provided in Table 1 (complete preprogram survey responses are provided in Multimedia Appendix 9). While all participants had previously performed PNBs using landmark or nerve stimulator techniques, none had previous experience with ultrasound guidance. Learning style preferences varied, although all participants favored workshops and hands-on clinical practice. Reported barriers included a lack of training opportunities and limited supplies.

**Table 1:** Baseline participant demographics (N=7).

Characteristic		Values
**Participant demographics**		
	Participants, n (%)	7 (100)
	Female, n (%)	4 (57.1)
	Experience (y), mean (SD)	9.1 (5.1)
**Have you used ultrasound previously? n (%)**		
	Yes	0 (0)
	No	7 (100)
**Do you have previous experience with blocks? n (%)**		
	Yes	7 (100)
	No	0 (0)
**What blocks have you performed?^a^ n (%)**		
	Supraclavicular	2 (28.6)
	Axillary	5 (71.4)
	Peripheral nerve (radial, medial, and ulnar)	4 (57.1)
	Ankle	5 (71.4)
	Sciatic	2 (28.6)
	Scalp	1 (14.2)
**What is your primary motivation for participating in this program?^a^ n (%)**		
	Learning new clinical skills	6 (85.7)
	Patient outcomes	5 (71.4)
	Work satisfaction	2 (28.6)
	Patient satisfaction	4 (57.1)
	Decreased resource use	3 (42.8)

^a^Multiple responses were allowed per participant.

Each participant completed the full online curriculum, attended both in-person workshops, and performed multiple supervised UGRA procedures during the clinical practice period. The number of blocks performed by each participant varied widely in the 10 months following implementation (range 14-41).

### Reaction Outcomes

The overall satisfaction scores for the program were high (Table 2). The most frequently cited strengths were the integration of theory with personalized hands-on practice. No component was identified as unhelpful; however, participants suggested increasing collaboration with surgical specialties and extending the duration of trainers on-site.

**Table 2:** Participants’ reactions to the training program.

Survey question	Score^a^, mean (SD)
Performing my own blocks during work hours was useful	4.71 (0.49)
The content of the program was satisfactory	4.86 (0.38)
The online study program helped prepare me for the in-person sessions	4.86 (0.38)
The conference presentations were useful	4.86 (0.38)
The program was applicable to my practice	5.00 (0)
The program should continue at HNC^b^	5.00 (0)
It was useful to practice with ultrasound during the workshops	5.00 (0)
The clinical teaching and supervision were helpful during procedures	5.00 (0)
The knowledge test was a fair evaluation of the course material	5.00 (0)
This program should be implemented in other hospitals	5.00 (0)

^a^Responses rated using a 5-point Likert scale (1=strongly disagree; 5=strongly agree).

^b^HNC: Hospital Nacional de Coatepeque.

### Learning Outcomes

**Figure 2:**
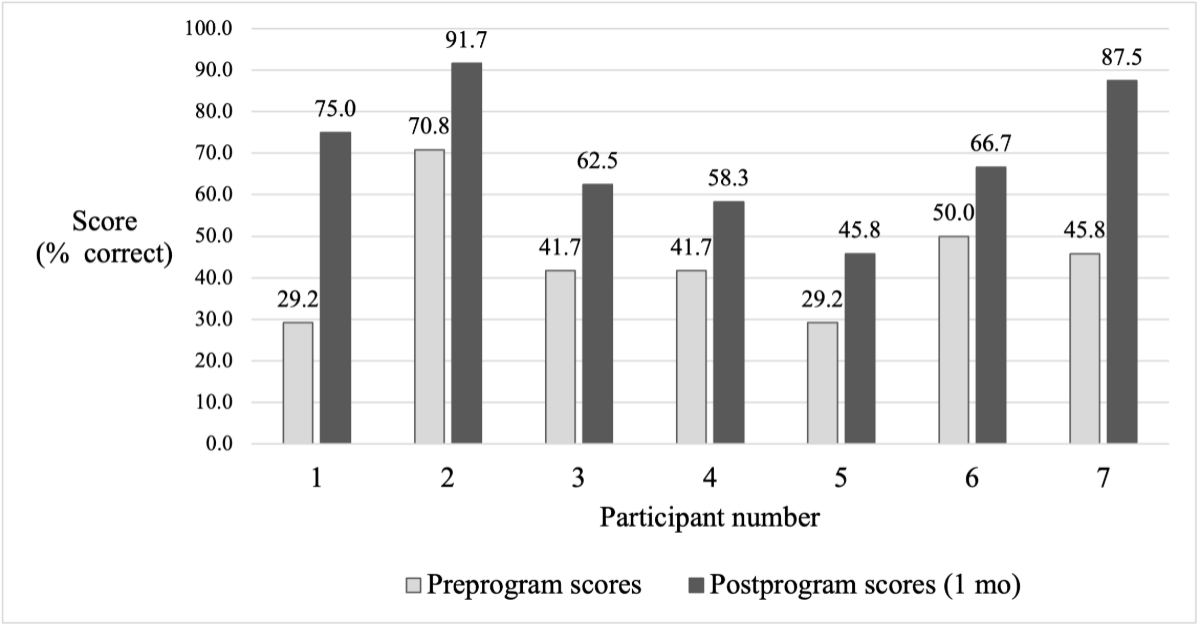
Participant knowledge test scores before training and 1 month after training.

**Figure 3:**
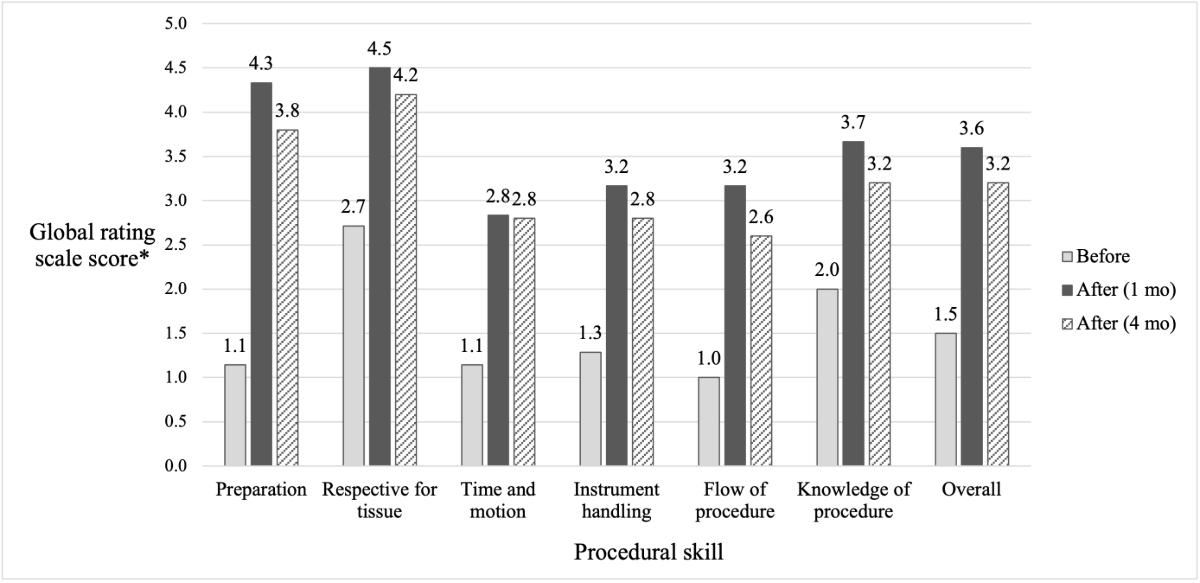
Change in procedural skills before and after the program at 1 and 4 months, measured by average global rating scale scores. *Using a 5-point scoring system (1=poor performance; 5=superior performance).

As shown in Figure 2, all participants demonstrated improved knowledge of UGRA. Knowledge test scores increased from a median of 45.8% (11/24; IQR 33.2%-54.0%) to 66.7% (16/24; IQR 58.3%-87.5%), representing a median absolute improvement of 20.8 (IQR 16.7%-41.7%; *P*=.02) percentage points with a large effect size (*r*=0.90, 95% CI 0.896-0.913). Similarly, all participants reported perceived gains in knowledge and confidence with UGRA (Table 3). Procedural skills improved following training across all domains, with median GRS scores increasing from 1.2 before the program to 3.4 at 1 month and 3.0 at 4 months (Figure 3). This corresponded to a median percentage increase of 147.1% (IQR 96.9%-197.3%; *P*=.03) at 1 month and 131.4% (IQR 90.5%-172.3%; *P*=.04) at 4 months, with large effect sizes at both time points (*r*=0.90, 95% CI 0.90-0.92 at 1 month; *r*=0.91, 95% CI 0.90-0.95 at 4 months). IRR for average preprogram scores showed moderate-to-high agreement with wide confidence intervals (intracorrelation coefficient=0.69, 95% CI 0.31-0.88; κ=0.63, 95% CI 0.47-0.93). The IRR for individual skills is provided in Multimedia Appendix 10, although the IRR could not be meaningfully calculated for some skills due to minimal variability. Only preprogram items had multiple raters due to the limited on-site availability of research personnel, providing preliminary evidence of measurement reliability. IRR was not assessed for postprogram items.

**Table 3:** Participants’ self-reported changes after participating in the training program.

Survey question	Score^a^, mean (SD)
I feel that I have improved my ability to perform blocks	4.29 (0.76)
My knowledge of PNBs^b^ has increased	4.43 (0.79)
I feel more comfortable using and interpreting ultrasound	4.57 (0.53)
I have a stronger desire to implement blocks in my practice	4.86 (0.38)
I plan to continue using PNBs in my practice	4.57 (0.53)
I feel that PNBs improve patient care at HNC^c^	5.00 (0)

^a^Responses rated using a 5-point Likert scale (1=strongly disagree; 5=strongly agree).

^b^PNB: peripheral nerve blocks.

^c^HNC: Hospital Nacional de Coatepeque.

### Behavior Outcomes

All participants reported high motivation to continue performing UGRA and intention to integrate PNBs into their practice (Table 3). All anesthesiologists continued to perform ultrasound-guided nerve blocks through the follow-up period after implementation.

### Clinical Outcomes

A total of 54 blocks were performed in the first month under direct supervision, and 187 total procedures were recorded during the 10 months following implementation (Table 4). Patient demographic and case information are provided in Multimedia Appendix 11. The overall procedure success rate was 96.3% (180/187), with no reported patient complications. The supraclavicular block was the most frequently performed (66/187, 35.3%). General anesthesia was avoided in 95.9% (70/73) of the patients for upper limb surgery through successful brachial plexus blocks. On the postprogram survey, all 7 anesthesiologists strongly agreed that UGRA improves patient care at HNC.

**Table 4:** Ultrasound-guided nerve blocks performed during the first 10 months after program implementation by type (n=187).

Block type	Blocks, n (%)
Interscalene	10 (5.3)
Supraclavicular	66 (35.3)
Femoral	35 (18.7)
Adductor canal	25 (13.4)
Popliteal	40 (21.4)
Other	11 (5.9)

## Discussion

### Principal Findings

We describe the successful design and implementation of a hybrid training program at a resource-limited hospital in southwestern Guatemala. This program resulted in substantial growth in knowledge and technical skills, practice change, and enhanced patient care for a 10-month period. The educational model developed for this context offers a framework for introducing UGRA services in LMICs where clinical capabilities are lacking.

### Educational Program Design

This program featured a unique educational setting, requiring input from local stakeholders to ensure alignment with daily clinical practice and local needs. Unlike previous initiatives, such as the original GRACE program, which aimed to expand preexisting UGRA capacity, anesthesiologists at HNC had no previous exposure to ultrasound or ultrasound-guided procedures. This lack of training presented challenges due to the steep learning curve and high-stakes environment in which these procedures are performed, reflecting the reality for much of the anesthesia workforce in LMICs [12]. Additionally, the paucity of available Spanish-language UGRA resources necessitated the translation or development of all surveys, lectures, and training materials in Spanish.

Multiple approaches have been used to deliver education in UGRA [27]. International programs have relied on either in-person training or predominantly online or virtual formats, with variable success [13,15,28-30]. Each method has inherent advantages and limitations. In-person training provides direct interaction with learners, hands-on skill development, real-time adaptation and feedback, and access to resources and equipment but is constrained by travel, time away from clinical duties, short program duration, and higher costs [31]. Conversely, online-only training offers flexibility, continuity, lower costs, and the ability to record and revisit content. However, it is limited by reliance on visual learning, time-zone variability, and internet connectivity and lacks in-person teaching and supervision. The duality of our methodology leverages the benefits of both approaches while minimizing the challenges and limitations.

Given the limited exposure to UGRA and trainer availability, a hybrid design was selected to maximize learning and skill development. Initial self-directed learning was used to establish foundational knowledge, particularly for theoretical concepts such as ultrasound physics, pharmacology, and management of complications. Concepts were then reinforced with classroom instruction and followed by workshops and clinical practice to support technical skill acquisition. This “flipped classroom” approach prepares learners for hands-on experiential learning and has become a standard in medical education [32]. Evidence suggests that combining self-directed learning with expert clinical instruction reduces procedural errors and improves performance [33,34]. Using this multifaceted approach, HNC anesthesiologists achieved proficiency and confidence with UGRA after 1 month and, importantly, maintained these skills even after direct trainer support had ended. Blended learning has been effectively integrated into other medical contexts [35-38]. Increasing access and familiarity with e-learning tools and resources will continue to expand opportunities for student-centered medical education worldwide.

### Impact on Patient Care

A notable impact of UGRA in this setting was the avoidance of general anesthesia for upper limb surgery through ultrasound-guided brachial plexus blocks. This was achieved in 70 (95.9%) of the 73 patients over the 10-month follow-up period. Regional anesthesia has well-documented advantages over general anesthesia, such as fewer perioperative respiratory events, reduced opioid use, superior pain management, and improvement in some procedure-specific outcomes [39]. These benefits are more pronounced in resource-limited settings, where constraints in supplies, equipment, and staffing reduce safety margins. Large datasets from Médecins Sans Frontières (Doctors Without Borders) facilities in LMICs similarly reported lower mortality with regional anesthesia in comparison to general endotracheal anesthesia [40]. Analgesic blocks were also performed during training and clinical practice and were perceived by both patients and surgeons to provide superior pain control compared with the existing nonopioid regimens, although formal evaluation is warranted. Nevertheless, all anesthesia participants unanimously agreed on the postprogram survey that PNBs improved patient care at their institution.

Patient safety was a priority for this pilot program. Ultrasound-based interventions require careful technique to achieve consistent and safe outcomes. Given that improper ultrasound application or technique may lead to unintended physiological effects or nerve injury, hands-on training becomes essential to ensure safe use [41,42]. In our program, no complications were observed, and the block failure rate of 3.7% was consistent or superior compared to published rates ranging from 5% to 10% [43-45].

### Lessons Learned

Implementing a regional anesthesia service at a resource-limited public hospital in Guatemala required adaptability and creative problem-solving. The preoperative workflow presented challenges, as patients typically arrived directly at the operative area with minimal preparation time, limiting the window for block placement and onset. Through pharmacological adjustments, the combination of the shorter-acting lidocaine with bupivacaine anecdotally accelerated the onset of blocks. Consent was typically not obtained by the anesthesia team; therefore, a new process was established to obtain authorization for procedures, especially parental consent in the case of minors, where parents were required to provide consent. In patients who were illiterate, we integrated the local protocol of reading the consent form information, allowing for questions, and then obtaining fingerprint stamps on forms. Technical limitations with portable ultrasound devices further complicated implementation. The prolonged procedure times during skill development led to probe overheating or battery depletion, and limited penetration of deeper structures hindered visualization in some blocks. The anesthesiologists’ unique 24-hour schedule every sixth day also required careful planning to ensure consistent practice and skill reinforcement. Despite variation in the number of blocks performed by trainees, consistent improvements in knowledge and skills were observed across all participants. Finally, establishing this new service necessitated collaboration with departments beyond anesthesiology and surgery to address issues involving supply chain, documentation processes, emergency preparedness protocols, postoperative monitoring, and nursing education on the wards.

Multiple strategic decisions contributed to the program’s success. First, the smaller cohort size, extended trainer presence on-site, and longer follow-up period increased direct interaction with experts, maximizing learning and enabling assessment of clinical practice and outcomes. Second, multidisciplinary coordination allowed for adjustment of the surgical schedule to dedicate one operating room to orthopedics, increasing block volume and creating greater opportunities for supervised practice. Third, limiting the focus to a few high-yield blocks proved effective for trainees with minimal baseline experience, as it allowed for focused application of learning and consolidation of skills. As proficiency grew with these limited blocks and ultrasound maneuvering, we then observed the adoption of new blocks among the group at HNC. Fourth, the development of data collection tools, such as the procedure log, assisted with real-time monitoring of progress, quality improvement, supply procurement, and research efforts. Finally, active engagement with hospital leadership and the Ministry of Public Health and Welfare, Guatemala not only reinforced the program’s value but also generated public recognition, laying the foundation for potential expansion across the national health system.

### Future Directions

While the initial results are encouraging, future efforts must continue to prioritize sustainability, ensuring progress is both durable and scalable. This includes implementing strategies for long-term follow-up, identifying and training local experts, and maintaining buy-in from local stakeholders [12]. Our group has engaged in regular communication and quarterly visits to reinforce relationships and provide mentorship. A long-term partnership with the CU Global Anesthesia program will include ongoing peer-to-peer support, faculty engagement, research collaborations, and resident rotations. Ultimately, the shared objective of this program is to develop local experts who can then train and support colleagues in the region. This “train-the-trainer” model has the potential to foster a self-sustaining system of local expertise and training cascades to build long-term capacity within the health system [46,47]. Efforts are underway to expand UGRA training to residency programs, equipping anesthesiologists early in their careers and integrating these techniques into both public and private practice, thereby expanding the scope and consistency of anesthesia care nationwide. Additionally, given the growing role of UGRA, future training initiatives could include collaboration with emergency physicians at HNC to integrate ultrasound-guided nerve block techniques into emergency care, allowing patients to benefit from regional anesthesia earlier in their clinical course.

Collaboration with local stakeholders, including nursing staff, surgeons, and hospital administration, is essential to the long-term success of this program. Hospital leadership engagement is critical to ensure the purchasing of supplies and medications needed to maintain a successful and safe UGRA program. To sustain interdisciplinary commitment, research should prioritize patient satisfaction, clinical outcomes, system-level impact, and cost analyses, thereby demonstrating value in patient care and resource optimization [48].

These data can also support efforts to increase access to ultrasound technology. Although donation-based models have helped bridge initial gaps, there remains a need for improved purchasing mechanisms and sustainable supply chains for portable devices in Guatemala and other Latin American countries where demand is high. Advances in technology continue to create new opportunities for training and patient safety, and recent studies demonstrate that technology-assisted models can enhance clinical decision-making and expand training capacity across multiple medical disciplines, even in resource-limited settings [49]. Technology-driven clinical training models increase accessibility, standardization, and clinical accuracy. This hybrid UGRA program, combining structured digital content with hands-on practice, offers a pragmatic approach to overcoming training barriers in LMICs.

### Limitations

There are multiple limitations to this study. First, training was delivered at a single hospital site with a small cohort of anesthesiologists. While this helped ensure quality control and exposure to practice with trainers, this limits generalizability to other departments, residency programs, or hospital systems. Second, there is a risk of information biases inherent in the observational study methods, such as recall or observer bias. Self-reporting may yield incomplete or inaccurate data; assessments of technical competency were performed by the training team with single raters for follow-up assessments, which may introduce expectancy and Hawthorne bias. Future studies should incorporate blinded, independent assessors or video adjudication to enhance objectivity and IRR. Additionally, although evaluation materials were adapted from validated sources, the translated GRACE instruments were not formally validated for cultural or psychometric equivalence, which may limit measurement accuracy. Program fidelity measures, such as detailed module completion analytics, were not available for all participants, which may limit precise quantification of exposure. However, attendance and active participation were confirmed for all anesthesiologists.

The costs of delivering in-person training can be prohibitive and should be considered when planning similar educational initiatives. Previous UGRA programs have reported costs up to US $5000 per trainer per trip [13]. This program minimized costs by integrating the hybrid educational model. Thus, costs were lower and estimated to be an average of US $1500 to US $2000 per trainer per trip. We believe that this model offers a pragmatic and cost-efficient approach for future international collaborations.

### Conclusions

This evaluation demonstrates the successful implementation of a hybrid training model for UGRA at a public hospital in Guatemala, advancing perioperative pain management in a resource-limited setting. By combining online modules, in-person didactics, and supervised clinical practice, the program achieved substantial improvements in anesthesiologists’ knowledge, procedural skills, and confidence. Despite a lack of previous capabilities, this program resulted in the adoption of UGRA, with sustained clinical practice observed over a 10-month follow-up period. These outcomes reflect both a tailored curriculum and strong local engagement, highlighting the model’s value, safety, and potential scalability in LMICs. The avoidance of general anesthesia in upper limb surgeries underscores UGRA’s potential to improve patient safety and optimize resources within the public health system. Future efforts should prioritize local expert development, interdisciplinary collaboration, and increased access to ultrasound devices to ensure the long-term sustainability and expansion of UGRA, thereby transforming anesthesiology care for underserved communities across Guatemala.

## Appendix

Multimedia Appendix 1 Preprogram participant survey.

Multimedia Appendix 2 Curriculum outline of online learning modules.

Multimedia Appendix 3 Outline of educational conference topics.

Multimedia Appendix 4 Postprogram participant survey.

Multimedia Appendix 5 Ultrasound-guided regional anesthesia knowledge test.

Multimedia Appendix 6 Global rating scale for procedural skills.

Multimedia Appendix 7 Procedure documentation form.

Multimedia Appendix 8 Procedure consent form.

Multimedia Appendix 9 Results of preprogram participant survey.

Multimedia Appendix 10 Interrater reliability for global rating scale assessment scores.

Multimedia Appendix 11 Characteristics of patients and procedures performed during the ultrasound-guided regional anesthesia training program.

## Abbreviations

CU: University of Colorado
GRACE: Global Regional Anesthesia Curricular Engagement
HNC: Hospital Nacional de Coatepeque
IRR: interrater reliability
LMIC: low- and middle-income country
PNB: peripheral nerve block
UGRA: ultrasound-guided regional anesthesia

## Data Availability

The datasets generated or analyzed during this study are available from the corresponding author on reasonable request.

## References

[1] Albrecht E, Chin KJ (2020). Advances in regional anaesthesia and acute pain management: a narrative review. Anaesthesia.

[2] Resta F, Barcella B, Angeli V, Lago E, Santaniello A, Dedato AS, Centurioni CE, Regeni E, Savastano S, Baldi E, Contri E, Maffeis R, Denti P, Musella V, Schicchi A, Lonati D, Salinaro F, Perlini S, Di Pietro S (2024). Simulation-based training in ultrasound-guided regional anaesthesia for emergency physicians: insights from an Italian pre/post intervention study. BMC Med Educ.

[3] Tucker RV, Peterson WJ, Mink JT, Taylor LA, Leech SJ, Nagdev AD, Leo M, Liu R, Stolz LA, Kessler R, Boulger CT, Situ-LaCasse EH, Avila JO, Huang R (2021). Defining an ultrasound-guided regional anesthesia curriculum for emergency medicine. AEM Educ Train.

[4] Di Pietro S, Maffeis R, Jannelli E, Mascia B, Resta F, De Silvestri A, Musella V, Centurioni CE, Regeni E, Grassi FA, Locatelli A, Perlini S (2025). Comparing the pericapsular nerve group block and fascia iliaca block for acute pain management in patients with hip fracture: a randomised clinical trial. Anaesthesia.

[5] Di Pietro S, Mascia B, Lo Bianco G, Perlini S, Iotti GA (2020). Anterior cutaneous nerve block for analgesia in anterior chest trauma: is the parasternal approach necessary?. Clin Exp Emerg Med.

[6] Di Pietro S, Caracciolo E, Barcella B, Perlini S, Regional Anaesthesia in Emergency Medicine Research Group (2022). Superficial cervical plexus block in emergency departments: rationale for its use in incision and drainage of neck skin abscesses. Intern Emerg Med.

[7] Schnittger T (2007). Regional anaesthesia in developing countries. Anaesthesia.

[8] Rukewe A, Fatiregun A (2010). The use of regional anesthesia by anesthesiologists in Nigeria. Anesth Analg.

[9] Ho M, Livingston P, Bould MD, Nyandwi JD, Nizeyimana F, Uwineza JB, Urquart R (2019). Barriers and facilitators to implementing a regional anesthesia service in a low-income country: a qualitative study. Pan Afr Med J.

[10] Admassie BM, Admass BA, Melesse DY (2024). Practice and challenges related to regional anesthesia in Amhara regional hospitals, Northwest-Ethiopia: a web-based survey study. BMC Anesthesiol.

[11] Ranger BJ, Bradburn E, Chen Q, Kim M, Noble JA, Papageorghiou AT (2023). Portable ultrasound devices for obstetric care in resource-constrained environments: mapping the landscape. Gates Open Res.

[12] Dohlman LE, Kwikiriza A, Ehie O (2020). Benefits and barriers to increasing regional anesthesia in resource-limited settings. Local Reg Anesth.

[13] Brouillette MA, Aidoo AJ, Hondras MA, Boateng NA, Antwi-Kusi A, Addison W, Singh S, Laughlin PT, Johnson B, Pakala SR (2020). Regional anesthesia training model for resource-limited settings: a prospective single-center observational study with pre-post evaluations. Reg Anesth Pain Med.

[14] Dohlman LE, Thakkar N, Jivanelli B, Pakala S, Brouillette MA, Global Regional Anesthesia and Pain Management Research Group (2022). Regional anesthesia global health collaborations- a scoping review of current intervention methods. Curr Opin Anaesthesiol.

[15] Liu M, Salmon M, Zaidi R, Nagdev A, Debebe F, Muller MF, Ruhangaza CF, Emiru H, Belachew Y, Tumebo A, Paoletti M, Okrainec A, Chan V, Niazi AU (2021). Ultrasound-guided regional anesthesia: feasibility and effectiveness of teaching via telesimulation in Ethiopia. Reg Anesth Pain Med.

[16] Avila C, Bright R, Gutierrez J, Hoadley K, Manuel C, Romero N, Rodriguez MP (2015). Guatemala health system assessment 2015. Health Finance & Governance Project.

[17] Zha Y, Truché P, Izquierdo E, Zimmerman K, de Izquierdo S, Lipnick MS, Law TJ, Gelb AW, Evans FM (2021). Assessment of anesthesia capacity in public surgical hospitals in Guatemala. Anesth Analg.

[18] Gamboa JE, Bolaños AG, Simmons CG (2025). Community perceptions of accessing surgical and anesthetic care in rural Guatemala: a cross-sectional survey. Cureus.

[19] Turner RA, Simmons CG, Ramirez S, Gamboa JE (2025). Hypoxemia and postoperative monitoring after anesthesia: a prospective observational study using portable pulse oximetry in a resource-limited setting in Guatemala. Cureus.

[20] Programa de estudio. Regional anestesia Guatemala.

[21] Kirkpatrick JD, Kirkpatrick WK (2016). Kirkpatrick's Four Levels of Training Evaluation.

[22] Anderson LN, Merkebu J (2024). The Kirkpatrick model: a tool for evaluating educational research. Fam Med.

[23] Chuan A, Graham PL, Wong DM, Barrington MJ, Auyong DB, Cameron AJ, Lim YC, Pope L, Germanoska B, Forrest K, Royse CF (2015). Design and validation of the Regional Anaesthesia Procedural Skills Assessment Tool. Anaesthesia.

[24] Gandhi K, Patel V, Maliakal T, Xu D, Flisinski K (2009). Universal documentation sheet for peripheral nerve blocks. J NY Sch Reg Anest.

[25] Rosenthal R (1991). Meta-Analytic Procedures for Social Research.

[26] Rosenthal R (1994). Science and ethics in conducting, analyzing, and reporting psychological research. Psychol Sci.

[27] Nix CM, Margarido CB, Awad IT, Avila A, Cheung JJ, Dubrowski A, McCartney CJ (2013). A scoping review of the evidence for teaching ultrasound-guided regional anesthesia. Reg Anesth Pain Med.

[28] Moll V, Schmidt PC, Amos C, Workneh RS, Tadesse M, Mulugeta E, Abate A (2021). Building regional anesthesia capacity in limited-resource settings: a pilot study evaluating a 4-week curriculum. Pain Manag.

[29] Burckett-St Laurent DA, Cunningham MS, Abbas S, Chan VW, Okrainec A, Niazi AU (2016). Teaching ultrasound-guided regional anesthesia remotely: a feasibility study. Acta Anaesthesiol Scand.

[30] Pfenninger EG, Tugtekin I, Stahl W, Dinse A, Gorsewski G, Vicent O, Sam-Awortwi W, Antwi-Kusi A, Himmelseher S (2018). “Anesthesia-focused sonography”: first analysis of transferring a training from Germany to Ghana. SDRP J Anesth Surg.

[31] Maloney S, Nicklen P, Rivers G, Foo J, Ooi YY, Reeves S, Walsh K, Ilic D (2015). A cost-effectiveness analysis of blended versus face-to-face delivery of evidence-based medicine to medical students. J Med Internet Res.

[32] Phillips J, Wiesbauer F (2022). The flipped classroom in medical education: a new standard in teaching. Trends Anaesth Crit Care.

[33] de Oliveira Filho GR, Mettrau FA (2018). The effect of high-frequency, structured expert feedback on the learning curves of basic interventional ultrasound skills applied to regional anesthesia. Anesth Analg.

[34] Udani AD, Macario A, Nandagopal K, Tanaka MA, Tanaka PP (2014). Simulation-based mastery learning with deliberate practice improves clinical performance in spinal anesthesia. Anesthesiol Res Pract.

[35] Koch R, Gassner L, Gerlach N, Festl-Wietek T, Hirt B, Joos S, Shiozawa T (2025). Integrated e-learning for shoulder anatomy and clinical examination skills in first-year medical students: randomized controlled trial. JMIR Med Educ.

[36] Vallée A, Blacher J, Cariou A, Sorbets E (2020). Blended learning compared to traditional learning in medical education: systematic review and meta-analysis. J Med Internet Res.

[37] Tudor Car L, Kyaw BM, Dunleavy G, Smart NA, Semwal M, Rotgans JI, Low-Beer N, Campbell J (2019). Digital problem-based learning in health professions: systematic review and meta-analysis by the digital health education collaboration. J Med Internet Res.

[38] Oftring ZS, Deutsch K, Tolks D, Jungmann F, Kuhn S (2025). Novel blended learning on artificial intelligence for medical students: qualitative interview study. JMIR Med Educ.

[39] Hughey S, Cole J, Drew B, Brust A, Stedjelarsen E (2025). Regional anesthesia in resource-limited and disaster environments: a daring discourse. Reg Anesth Pain Med.

[40] Ariyo P, Trelles M, Helmand R, Amir Y, Hassani GH, Mftavyanka J, Nzeyimana Z, Akemani C, Ntawukiruwabo IB, Charles A, Yana Y, Moussa K, Kamal M, Suma ML, Ahmed M, Abdullahi M, Wong EG, Kushner A, Latif A (2016). Providing anesthesia care in resource-limited settings: a 6-year analysis of anesthesia services provided at Médecins Sans Frontières facilities. Anesthesiology.

[41] Ozsoy U, Ogut E, Sekerci R, Hizay A, Rink S, Angelov DN (2019). Effect of pulsed and continuous ultrasound therapy on the degree of collateral axonal branching at the lesion site, polyinnervation of motor end plates, and recovery of motor function after facial nerve reconstruction. Anat Rec (Hoboken).

[42] El-Tallawy SN, Ahmed RS, Salem GI, Alzahrani TA, Haddara MM, Ahmed RH, Nagiub MS, Alsubaie AT, Ali MM, Elbasha MM, Ahmed AA (2025). Neurological deficits following regional anesthesia and pain interventions: reviewing current standards of care. Pain Ther.

[43] Barrington MJ, Watts SA, Gledhill SR, Thomas RD, Said SA, Snyder GL, Tay VS, Jamrozik K (2009). Preliminary results of the Australasian Regional Anaesthesia Collaboration: a prospective audit of more than 7000 peripheral nerve and plexus blocks for neurologic and other complications. Reg Anesth Pain Med.

[44] Bottomley T, Gadsden J, West S (2023). The failed peripheral nerve block. BJA Educ.

[45] Cotter JT, Nielsen KC, Guller U, Steele SM, Klein SM, Greengrass RA, Pietrobon R (2004). Increased body mass index and ASA physical status IV are risk factors for block failure in ambulatory surgery - an analysis of 9,342 blocks. Can J Anaesth.

[46] Mormina M, Pinder S (2018). A conceptual framework for training of trainers (ToT) interventions in global health. Global Health.

[47] Anderson CR, Taira BR (2018). The train the trainer model for the propagation of resuscitation knowledge in limited resource settings: a systematic review. Resuscitation.

[48] Mohamed SS, Temu R, Komba LF, Kaino MM, Olotu FI, Ndebea AS, Vaughan BN (2024). Patient satisfaction with, and outcomes of, ultrasound-guided regional anesthesia at a referral hospital in Tanzania: a cross-sectional study. Anesth Analg.

[49] Ogut E (2025). Artificial intelligence in clinical medicine: challenges across diagnostic imaging, clinical decision support, surgery, pathology, and drug discovery. Clin Pract.

