# Supplementary material for: Ultrasound-Guided Regional Anesthesia in a Resource-Limited Hospital: Prospective Pilot Study of a Hybrid Training Program
Source: JMIR Med Educ. 2026 Jan 8;12:e84181. doi: 10.2196/84181 (PMC12828311; doi:10.2196/84181)
Supplement: Multimedia Appendix 1 [file mededu_v12i1e84181_app1.docx]

**Multimedia Appendix 1: Pre-Program Survey**

1. Have you previously used ultrasound?
   1. Yes
   2. No
2. Do you have any prior experience performing peripheral nerve blocks?
   1. Yes
   2. No
3. If Yes to #2, what type of nerve block have you performed? (select all that apply)
   1. Interscalene
   2. Supraclavicular
   3. Infraclavicular
   4. Axillary
   5. Ulnar/Median/Radial
   6. Finger
   7. Femoral
   8. Adductor canal (saphenous)
   9. Sciatic (subgluteal)
   10. Sciatic (popliteal)
   11. Ankle
   12. Head/scalp
   13. Fascial plane blocks
4. If Yes to #2, what technique did you use?
   1. Nerve stimulation
   2. Surface anatomy-based landmarks
   3. Perivascular/transvascular
   4. Ultrasound
5. Is regional anesthesia relevant to your practice?
   1. Yes
   2. No
6. Which type of nerve block would be most useful in your practice? (select all that apply)
   1. Shoulder
   2. Upper arm
   3. Distal arm
   4. Upper leg
   5. Lower leg
7. Were peripheral nerve blocks taught during your residency training?
   1. Yes
   2. No
8. Where have you learned most about regional anesthesia?
   1. Didactic lectures
   2. Online training program
   3. Independent learning with online resources
   4. Peer reviewed journals
   5. Textbooks
9. What is your preferred learning style?
   1. Reading online resources
   2. Reading textbooks
   3. Reading peer-reviewed journals
   4. Online/in-person lectures
   5. Hands-on training/workshops
10. What is your primary motivation for taking this course? (Select all that apply)
    1. Learn new clinical skills
    2. Job satisfaction
    3. Patient outcomes
    4. Patient satisfaction
    5. Decreased resource utilization
    6. Other: ___________
11. What are the biggest challenges to performing nerve blocks in Guatemala? (Select all that apply)
    1. Lack of training opportunities
    2. Lack of supplies (ultrasound, needles, etc)
    3. Lack of personnel/time
    4. Patient refusal/beliefs

**Encuesta previa al programa**

1. ¿Ha utilizado ultrasonido anteriormente?
   1. Sí
   2. No
2. ¿Tiene alguna experiencia previa realizando bloqueos de nervios?
   1. Sí
   2. No
3. Si respondió Sí a la pregunta 2, ¿qué tipo de bloqueo de nervio ha realizado? (seleccione todas las que correspondan)
   1. Interescalénico
   2. Supraclavicular
   3. Infraclavicular
   4. Axilar
   5. Cubital/Mediana/Radial
   6. Dedo
   7. Femoral
   8. Canal aductor (safenoso)
   9. Ciático (subglúteo)
   10. Ciático (poplíteo)
   11. Tobillo
   12. Cabeza/cuero cabelludo
   13. Bloqueos del plano fascial
4. Si respondió Sí a la pregunta 2, ¿qué técnica utilizó?
   1. Estimulación nerviosa
   2. Puntos de referencia basados en la anatomía de la superficie
   3. Perivascular/transvascular
   4. Ultrasonido
5. ¿Es la anestesia regional aplicable a su práctica?
   1. Sí
   2. No
6. ¿Qué tipo de bloqueo de nervio sería más útil? (seleccione todas las que correspondan)
   1. Hombro
   2. Parte superior del brazo
   3. Brazo distal
   4. Muslo
   5. Pierna
7. ¿Se enseñaron bloqueos de nervios periféricos durante su residencia?
   1. Sí
   2. No
8. ¿Dónde más ha aprendido sobre la anestesia regional?
   1. Conferencias didácticas
   2. Programa de formación en línea
   3. Aprendizaje independiente con recursos en línea.
   4. Revistas revisadas por colegas
   5. Libros de texto
9. ¿Cuál es su método de aprendizaje preferido?
   1. Leer recursos en línea
   2. Leer libros de texto
   3. Leer revistas revisadas por pares
   4. Conferencias en línea/presenciales
   5. Talleres/formación práctica
10. ¿Qué es lo que más le motiva a participar en este curso? (Seleccione todas las que correspondan)
    1. Aprende nuevas habilidades clínicas
    2. Satisfacción laboral
    3. Resultados de los pacientes
    4. Satisfacción del paciente
    5. Disminución de la utilización de recursos
    6. Otro: ___________
11. ¿Cuáles son los mayores desafíos para realizar bloqueos de nervios en Guatemala? (Seleccione todas las que correspondan)
    1. Falta de oportunidades de formación.
    2. Falta de insumos (ultrasonido, agujas, etc)
    3. Falta de personal/tiempo
    4. Creencias/preferencias del paciente

This is a Multimedia Appendix to a full manuscript published in the J Med Internet Res. For full copyright and citation information see http://dx.doi.org/10.2196/jmir.84181
