# Supplementary material for: Ultrasound-Guided Regional Anesthesia in a Resource-Limited Hospital: Prospective Pilot Study of a Hybrid Training Program
Source: JMIR Med Educ. 2026 Jan 8;12:e84181. doi: 10.2196/84181 (PMC12828311; doi:10.2196/84181)
Supplement: Multimedia Appendix 2 [file mededu_v12i1e84181_app2.docx]

Multimedia Appendix: Ultrasound-Guided Regional Anesthesia Online Curriculum Outline

Visit online at: [https://anestesiaregionalguatemala.com](https://anestesiaregionalguatemala.com/)

**Week 1: Preparation and Pharmacology**

*Objectives:*

- Review considerations for preparation and monitoring during peripheral nerve blocks.
- Describe the components of the pre-procedure checklist.
- Review the pharmacology of the different local anesthetics used for blocks.
- Calculate maximum doses of local anesthetics.

*Modules*

1. Set-up and Safety
2. Pharmacology of Local Anesthetics

**Week 2: Blocks of the Upper Extremity**

*Objectives*:

- Describe the indications, technique, and complications of the interscalene block.
- Describe the indications, technique, and complications of the supraclavicular block.
- Review the relevant anatomy and ultrasound images obtained during these blocks.

*Modules*:

1. Interscalene Block
2. Supraclavicular Block

**Week 2: Blocks of the Lower Extremity**

*Objectives*:

- Describe the indications, technique, and complications of the femoral nerve block.
- Describe the indications, technique, and complications of the adductor canal nerve block.
- Describe the indications, technique, and complications of the popliteal block.
- Review the relevant anatomy and ultrasound images obtained during these blocks.

*Modules*:

1. Femoral and Adductor Canal Blocks
2. Popliteal Block (Sciatic Nerve)

**Week 4: Ultrasound Image Optimization and Management of Complications**

*Objectives*:

- Describe the functions, adjustments, and techniques used with ultrasound to optimize visualization.
- Review the mechanisms of nerve injury and strategies to prevent it.
- Review the mechanism, presentation, prevention, and management of local anesthetic systemic toxicity.

*Modules*:

1. Ultrasound Image Optimization
2. Complications and Management

This is a Multimedia Appendix to a full manuscript published in the J Med Internet Res. For full copyright and citation information see http://dx.doi.org/10.2196/jmir.84181
