# Supplementary material for: Ultrasound-Guided Regional Anesthesia in a Resource-Limited Hospital: Prospective Pilot Study of a Hybrid Training Program
Source: JMIR Med Educ. 2026 Jan 8;12:e84181. doi: 10.2196/84181 (PMC12828311; doi:10.2196/84181)
Supplement: Multimedia Appendix 3 [file mededu_v12i1e84181_app3.docx]

**Ultrasound-Guided Regional Anesthesia Guatemala 2024: Conference Agenda**

Learning objectives:

1. Discuss preparation, set-up, and considerations for informed consent and monitoring.
2. Review basic ultrasound physics, settings, maneuvering, and interpretation.
3. Describe the different types of nerve blocks’ indications, coverage, technique, and complications.
4. Review basic pharmacology and utilization of local anesthetics for nerve blocks.
5. Understand perioperative nursing considerations.
6. Practice ultrasound maneuvering, structure identification, and needle guidance.
7. Observe clinical demonstration of ultrasound-guided nerve blocks.
8. Review complications and management of emergencies.

Program outline

**Day 1:**

- Introduction and opening remarks
- Lecture 1: Understanding the role that US guided peripheral nerve blocks have in improving patient outcomes and satisfaction
- Lecture 2: The ultrasound machine: physics, manipulation, and utilization
- Lecture 3: The basics: set up and preparation
- Lecture 4: Perioperative nursing considerations: What to expect in the pre-op holding area
- Lecture 5: Peripheral nerve blocks for upper extremity surgery
- Sessions: Workshops and simulation

**Day 2:**

- Introduction
- Clinical demonstration
- Lecture 1: Pharmacology and agent selection
- Lecture 2: Local Anesthetic Systemic Toxicity: identification, management, prevention
- Lecture 3: Peripheral nerve blocks for lower extremity surgery
- Lecture 4: Perioperative nursing considerations: What to expect in the Post Anesthesia Care Unit
- Lecture 5: The Acute Pain Service: creating a dedicated team to combat pain and improve patient quality
- Lecture 6: Future directions
- Sessions: Workshops & simulations

This is a Multimedia Appendix to a full manuscript published in the J Med Internet Res. For full copyright and citation information see http://dx.doi.org/10.2196/jmir.84181
