# Supplementary material for: Ultrasound-Guided Regional Anesthesia in a Resource-Limited Hospital: Prospective Pilot Study of a Hybrid Training Program
Source: JMIR Med Educ. 2026 Jan 8;12:e84181. doi: 10.2196/84181 (PMC12828311; doi:10.2196/84181)
Supplement: Multimedia Appendix 4 [file mededu_v12i1e84181_app4.docx]

Cuestionario post-programa

Para cada una de las siguientes afirmaciones, indique su nivel de acuerdo.

|  | | 1 - Muy en desacuerdo | 2- En desacuerdo | 3- Neutral | 4- De acuerdo | 5- Muy de acuerdo |
| --- | --- | --- | --- | --- | --- | --- |
| El programa era aplicable a mi práctica. |  | |  |  |  |  |
| El programa debería continuar en el HNC. |  | |  |  |  |  |
| El contenido del programa fue satisfactorio. |  | |  |  |  |  |
| El programa de estudios en línea fue útil para prepararme para la conferencia práctica. |  | |  |  |  |  |
| Las presentaciones de la conferencia fueron útiles. |  | |  |  |  |  |
| Fue útil poder practicar con ultrasonido durante los talleres de la conferencia. |  | |  |  |  |  |
| Fue útil poder realizar mis propios bloqueos durante la semana. |  | |  |  |  |  |
| Me ayudaron la formación clínica y la supervisión durante los procedimientos en pacientes. |  | |  |  |  |  |
| El examen de conocimientos fue una evaluación justa de la información impartida. |  | |  |  |  |  |
| Creo que este programa debería implementarse en otros hospitales en Guatemala. |  | |  |  |  |  |

¿Cuál cree que fue la parte más útil de este programa?

________________________________________________________________

¿Cuál cree que fue la parte menos útil del programa?

________________________________________________________________

¿Qué sugerencias tiene para mejorar el programa?

________________________________________________________________

Para cada una de las siguientes afirmaciones, indique su nivel de acuerdo en una escala del 1 al 5.

1 = Muy en desacuerdo, 2 = En desacuerdo, 3 = Neutral, 4 = De acuerdo, 5 = Muy de acuerdo

|  | 1 | 2 | 3 | 4 | 5 |
| --- | --- | --- | --- | --- | --- |
| Me siento más cómodo/a usando e interpretando el ultrasonido. |  |  |  |  |  |
| Mis conocimientos sobre bloqueos de nervios periféricos han aumentado. |  |  |  |  |  |
| Siento que he mejorado mi habilidad de realizar bloqueos de nervios guiados por ultrasonido. |  |  |  |  |  |
| Ahora tengo un deseo más fuerte de implementar bloqueos de nervios en mi práctica. |  |  |  |  |  |
| Planeo continuar realizando bloqueos de nervios en mi práctica |  |  |  |  |  |
| Siento que los bloqueos de nervios mejoran la atención al paciente en HNC. |  |  |  |  |  |

This is a Multimedia Appendix to a full manuscript published in the J Med Internet Res. For full copyright and citation information see http://dx.doi.org/10.2196/jmir.84181
