# Supplementary material for: Ultrasound-Guided Regional Anesthesia in a Resource-Limited Hospital: Prospective Pilot Study of a Hybrid Training Program
Source: JMIR Med Educ. 2026 Jan 8;12:e84181. doi: 10.2196/84181 (PMC12828311; doi:10.2196/84181)
Supplement: Multimedia Appendix 5 [file mededu_v12i1e84181_app5.docx]

**Knowledge Test (English)**

*Adapted from: Brouillette et al [1] for content and language*

**Key:**

a: Modified question from original instrument

b: New test question

Please select the one best answer for each question. Do not leave any questions blank.

1) Which nerves are posterior and lateral to the popliteal artery in this image?^a^


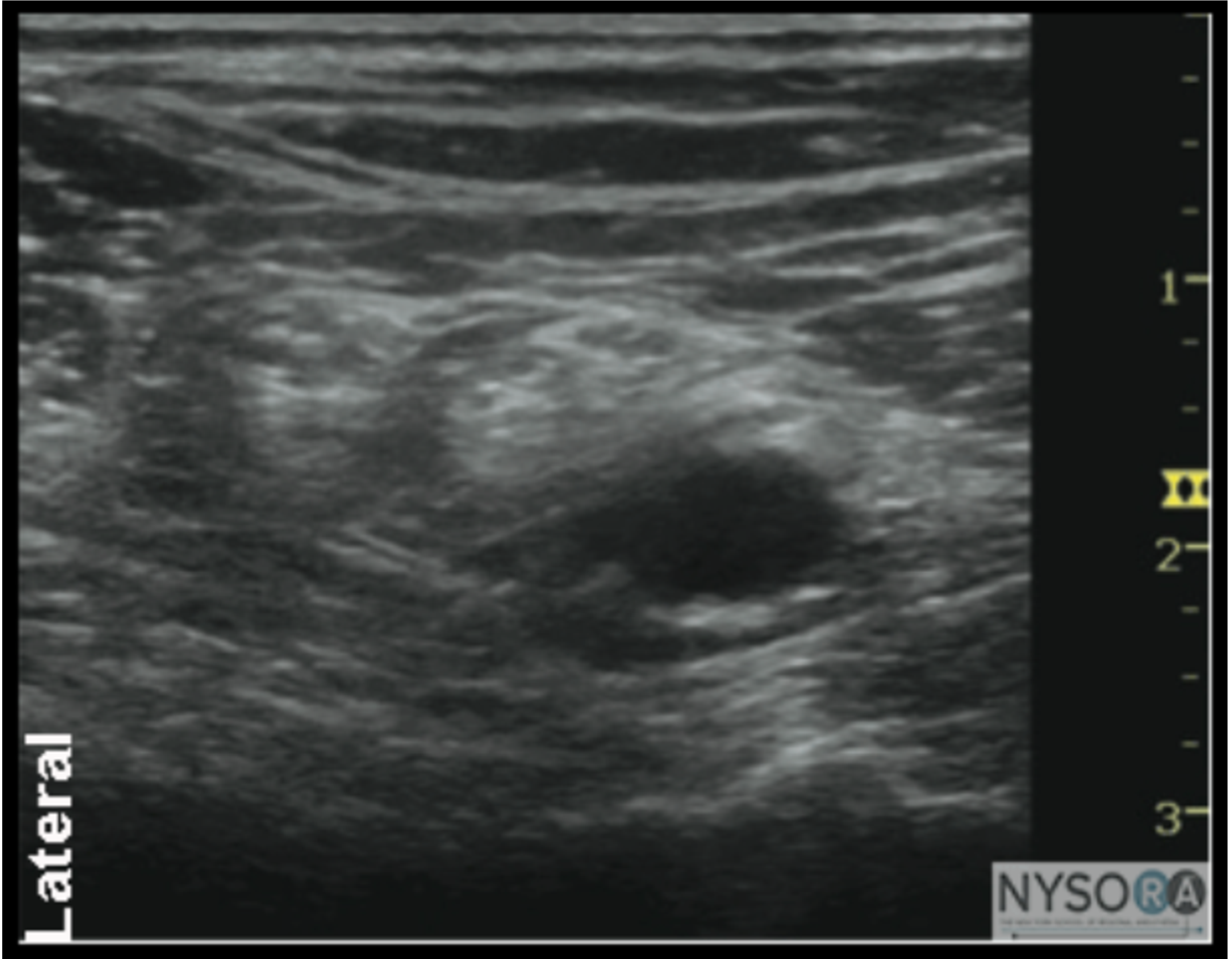


[Image accessed 1/28/24 from www.nysora.com]

1. Common peroneal and tibial
2. Common peroneal and saphenous
3. Sciatic and saphenous
4. Tibial and sural

2) What nerve block would provide the best anesthesia for foot surgery?

A. Common peroneal

B. Sciatic

C. Tibial

D. Saphenous

3) Which ultrasound mode is commonly used to visualize flow of blood through a vessel during performance of a nerve block?

A. Pulse-wave doppler mode

B. Amplitude mode

C. Color doppler mode

D. Continuous-wave doppler mode

4) Which of the following is not a necessary component of informed consent for a peripheral nerve block procedure?

A. Presentation of most harmful risks

B. Presentation of anticipated benefits

C. Presentation of most common risks

D. Patient signature that protects the physician against malpractice claims

5) What part of the upper limb is not anesthetized by any of the brachial plexus blocks?

A. Lateral portion of the forearm

B. Medial portion of the forearm

C. Medial portion of upper arm

D. Skin overlying the deltoid muscle

6) How does local anesthetic systemic toxicity typically present, from early to late stages?^a^

A. Paresthesia of the mouth and tongue → drowsiness → respiratory arrest → cardiac arrest → seizure

B. Drowsiness → seizure → paresthesias of the mouth and tongue → cardiac arrest → respiratory arrest

C. Drowsiness → paresthesias of the mouth and tongue → seizure → respiratory arrest → cardiac arrest

D. Respiratory arrest → drowsiness → paresthesias of the mouth and tongue → seizure → cardiac arrest

7) Which of the following is not a recommended monitor for performance of a peripheral nerve block?

A. Pulse oximeter

B. Thermometer

C. Clinical assessment of mental status

D. Non-invasive blood pressure monitor

8) Which of the following is the best way to confirm local anesthetic is not being injected intravascularly?^a^

A. Negative aspiration

B. Ultrasound visualization of needle tip outside of vasculature

C. Visualization of local anesthetic spread outside of vasculature on ultrasound image

D. All of the above

9) Which of the following is an advantage of an adductor canal block compared to a femoral block?^b^

1. Superior analgesia of anterior thigh
2. Greater distal blockade of lower leg
3. Less quadriceps motor block
4. Decreased risk of toxicity

10) When equipotent doses are used, which local anesthetic has the longest duration of action when used for peripheral nerve blockade?^a^

A. Lidocaine

B. Bupivacaine

C. Mepivicaine

D. Chloroprocaine

11) Ultrasound machines do not produce quality images of structures opposite of which of the following tissues?

A. Blood and fluid

B. Bone and air

C. Cartilage

D. Muscle

12) All of the following are hypothesized mechanisms by which nerves can be damaged during peripheral nerve block, except:

A. Inflammatory reaction

B. Disruption of lymphatic drainage by large volume of local anesthetic medication

C. Mechanical trauma caused by block needle

D. Direct neurotoxicity from injected local anesthetic medication

13) What is the most common complication of the supraclavicular approach to brachial plexus block?

A. Pneumothorax

B. Hoarse voice

C. Blockade of the phrenic nerve

D. Horner’s syndrome

14) What structure is immediately medial to the femoral nerve in this image?


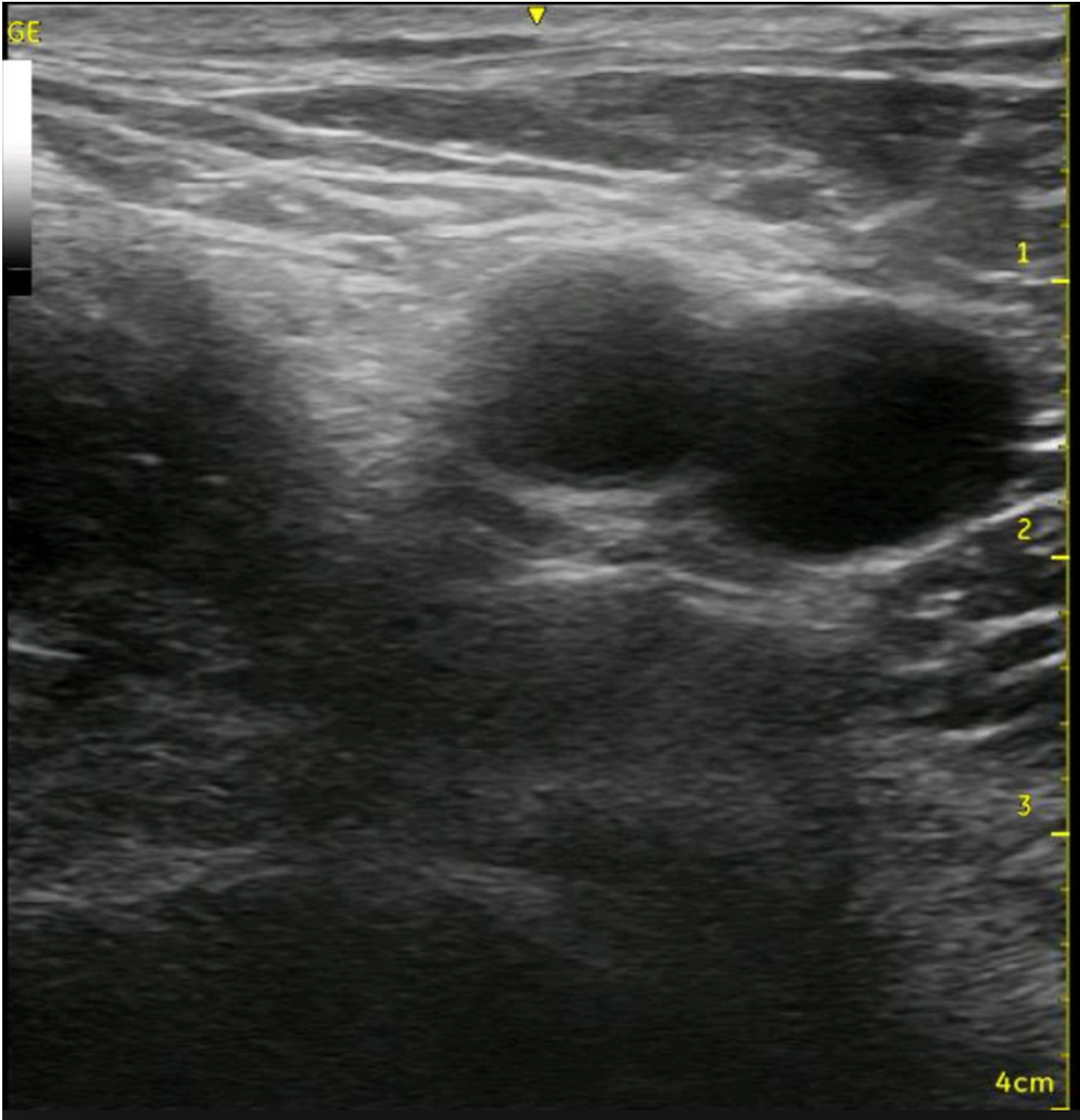


[Image accessed 1/28/24 from http://cpnbconsulting.com/wp-content/uploads/2012/04/Femoral-pic.jpg]

1. Femoral artery
2. Femoral vein
3. Lymphatics
4. Iliopsoas muscle

15) What approach to brachial plexus nerve block is shown in this image?


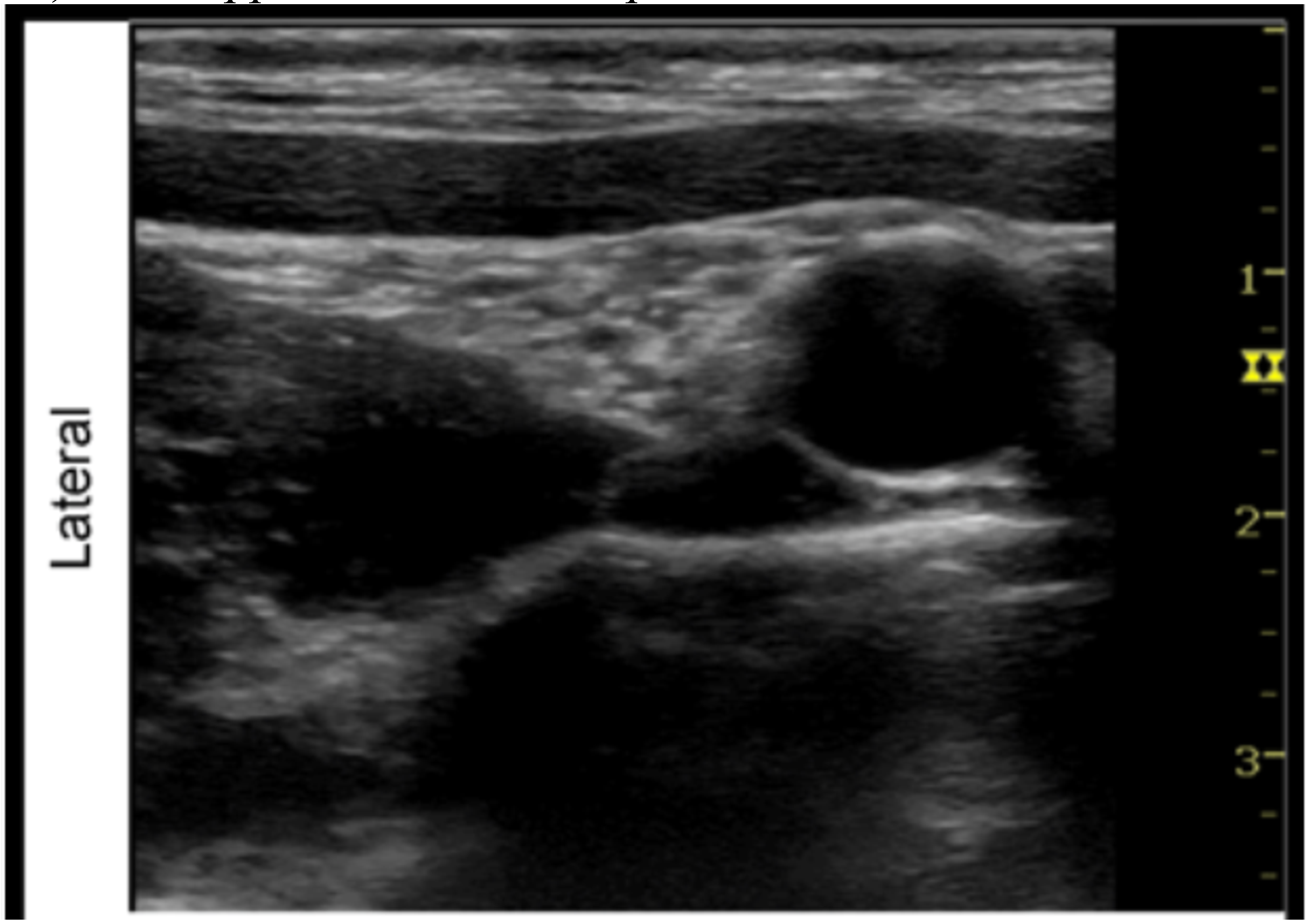


[Image accessed 1/28/24 from https://accessanesthesiology.mhmedical.com/data/books/hadzpnb2/m_hadzpnb2_c030f003.png]

1. Axillary
2. Infraclavicular
3. Supraclavicular
4. Interscalene

16) True or false: Most neuropraxias caused by peripheral nerve blocks resolve spontaneously.^a^

A. True

B. False

17) A 70 kilogram male is recovering from open reduction and internal fixation of bilateral tibial fractures done under general anesthesia. The surgeon and patient request your help with pain control, and you offer to perform bilateral sciatic nerve blocks. What is the recommended maximum dose of 0.5% bupivacaine for this patient?

A. 25 milliliters

B. 35 milliliters

C. 45 milliliters

D. 55 milliliters

18) In addition to usual basic and advanced life support measures, what treatment should also be incorporated into management of life-threatening local anesthetic systemic toxicity (LAST)?

A. Norepinephrine

B. Propofol

C. Vasopressin

D. Intralipid (20% fat emulsion)

19) What is one advantage of the supraclavicular approach to brachial plexus block as compared to the interscalene approach?

A. The medial aspect of the hand is more reliably anesthetized

B. The risk of pneumothorax is lower

C. The site is more compressible and compliant should bleeding occur

D. The technique is easier to perform

20) Which nerve is not a branch of the sciatic nerve?^a^

A. Common peroneal

B. Posterior tibial

C. Sural

D. Saphenous

21) What part of the lower limb may not be anesthetized by a femoral nerve block?^a^

A. The anterior thigh

B. The medial lower leg

C. The quadriceps muscle

D. Skin overlying the lateral thigh

22) What part of the lower limb is anesthetized by a femoral nerve block?

A. Medial portion of the lower leg

B. Sole of the foot

C. Posterior aspect of the thigh

D. Lateral ankle and foot

23) The nerve roots in the interscalene view can typically be found between which two structures?^b^

A. Anterior and middle scalene muscles

B. Posterior and middle scalene muscles

C. Anterior scalene and sternocleidomastoid muscles

D. Middle scalene and sternocleidomastoid muscles

24) What is the initial bolus dose of lipid emulsion to give a patient to treat local anesthetic toxicity?^b^

A. 0.25 mL/kg

B. 1.5 mL/kg

C. 5 mL/kg

D. 7.5 mL/kg

**Examen de Conocimiento**

1) ¿Qué nervios están posteriores y laterales a la arteria poplítea en esta imagen?


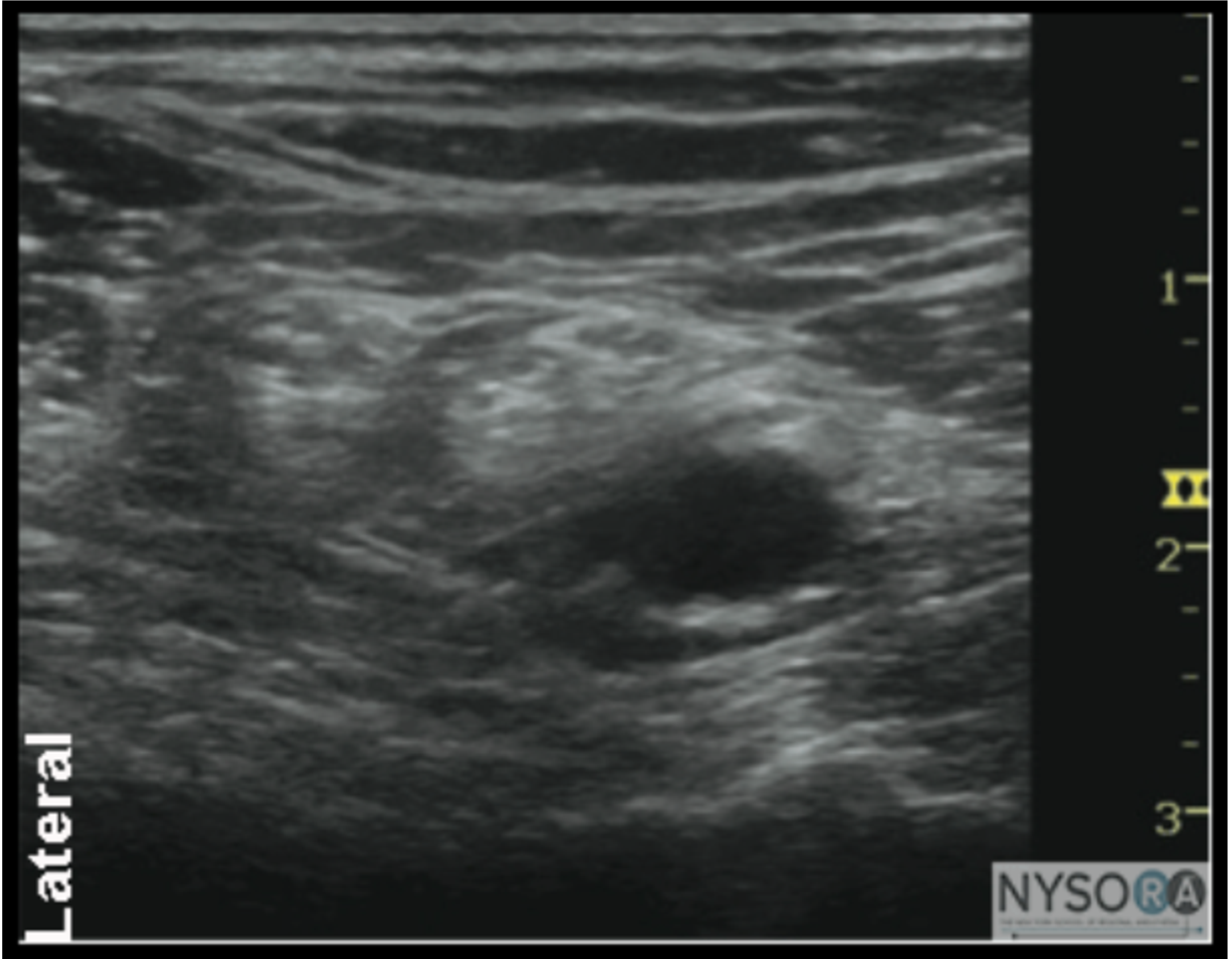


[Image accessed 1/28/24 from www.nysora.com]

A. Peroneo común y tibial

B. Peroneo común y safeno

C. Ciático y safeno

D. Tibial y sural

2) ¿Qué bloqueo de nervio proporcionaría la mejor anestesia para la cirugía del pie?

A. Peroneo común

B. Ciático

C. Tibial

D. Safeno

3) ¿Qué modo de ultrasonido se usa comúnmente para visualizar el flujo de sangre a través de un vaso durante la realización de un bloqueo de nervio?

A. Modo Doppler de onda de pulso

B. Modo de amplitud

C. Modo Doppler color

D. Modo Doppler de onda continua

4) ¿Cuál de los siguientes no es un componente necesario del consentimiento informado para un procedimiento de bloqueo de nervio periférico?

A. Presentación de los riesgos más perjudiciales

B. Presentación de los beneficios previstos

C. Presentación de los riesgos más comunes

D. Firma del paciente que protege al médico contra reclamaciones por negligencia

5) ¿Qué parte del miembro superior no se anestesia con ninguno de los bloqueos del plexo braquial?

A. Porción lateral del antebrazo

B. Porción medial del antebrazo

C. Porción medial de la parte superior del brazo

D. Piel que recubre el músculo deltoides

6) ¿Cómo se presenta típicamente la toxicidad sistémica de los anestésicos locales, desde las etapas tempranas hasta las tardías?

A. Parestesia de la boca y la lengua → somnolencia → paro respiratorio → paro cardíaco → convulsiones

B. Somnolencia → convulsiones → parestesias de la boca y la lengua → paro cardíaco → paro respiratorio

C. Parestesia de la boca y la lengua → somnolencia → convulsiones → paro respiratorio → paro cardíaco

D. Paro respiratorio → somnolencia → parestesias de la boca y la lengua → convulsiones → paro cardíaco

7) ¿Cuál de los siguientes no es un monitor recomendado para realizar un bloqueo de nervio periférico?

A. Oxímetro de pulso

B. Termómetro

C. Evaluación clínica del estado mental

D. Monitor de presión arterial no invasivo

8) ¿Cuál de las siguientes es la mejor manera de confirmar que el anestésico local no se está inyectando por vía intravascular?

A. Aspiración negativa

B. Visualización ecográfica de la punta de la aguja fuera de la vasculatura

C. Visualización de la propagación del anestésico local fuera de la vasculatura en una imagen de ultrasonido

D. Todo lo anterior

9) ¿Cuál de las siguientes es una ventaja de un bloqueo safeno en comparación con un bloqueo femoral?

A. Mejor analgesia de la parte anterior del muslo

B. Mejor analgesia de la parte inferior de la pierna

C. Menos bloqueo motor del cuádriceps

D. Disminución del riesgo de toxicidad

10) Cuando se utilizan dosis equipotentes, ¿cuál anestésico tiene la mayor duración de acción cuando se utiliza para el bloqueo de nervios periféricos?

A. lidocaína

B. bupivacaína

C. mepivacaíne

D. cloroprocaína

11) ¿Las máquinas de ultrasonido no producen imágenes de calidad de estructuras opuestas a cuál de los siguientes tejidos?

A. Sangre y líquido

B. Hueso y aire

C. Cartílago

D. Músculo

12) Todos los siguientes son mecanismos hipotéticos por los cuales los nervios pueden dañarse durante el bloqueo de nervios periféricos, excepto:

A. Reacción inflamatoria

B. Interrupción del drenaje linfático por un gran volumen de medicación anestésica local.

C. Trauma mecánico causado por bloqueo de aguja.

D. Neurotoxicidad directa por medicación anestésica local inyectada

13) ¿Cuál es la complicación más común del abordaje supraclavicular para el bloqueo del plexo braquial?

A. Neumotórax

B. Voz ronca

C. Bloqueo del nervio frénico

D. Síndrome de Horner

14) ¿Qué estructura está inmediatamente medial al nervio femoral en esta imagen?
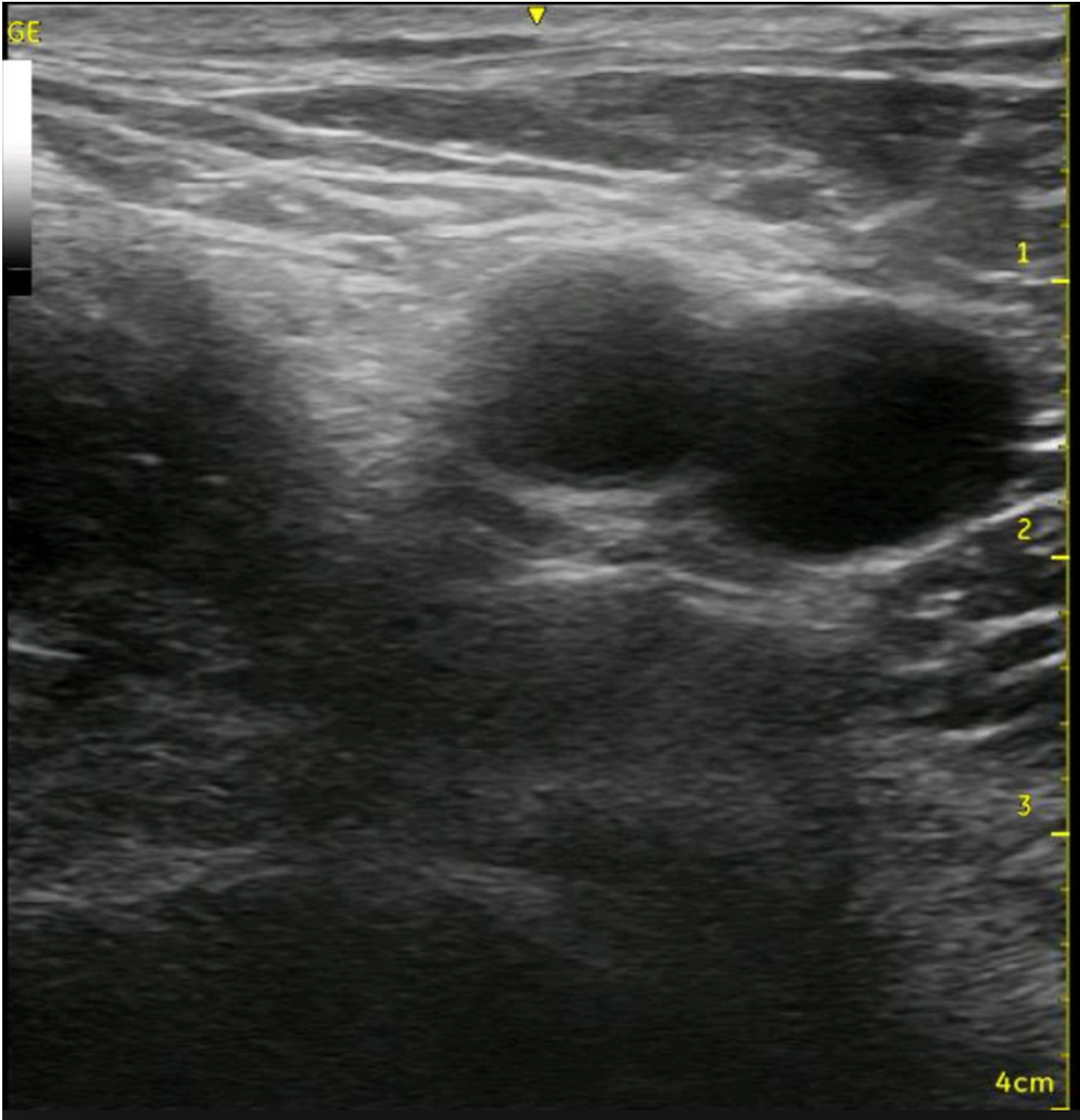


[Image accessed 1/28/24 from http://cpnbconsulting.com/wp-content/uploads/2012/04/Femoral-pic.jpg]a

A. Arteria femoral

B. Vena femoral

C. Linfáticos

D. Músculo iliopsoas

15) ¿Qué abordaje para el bloqueo del nervio del plexo braquial se muestra en esta imagen?


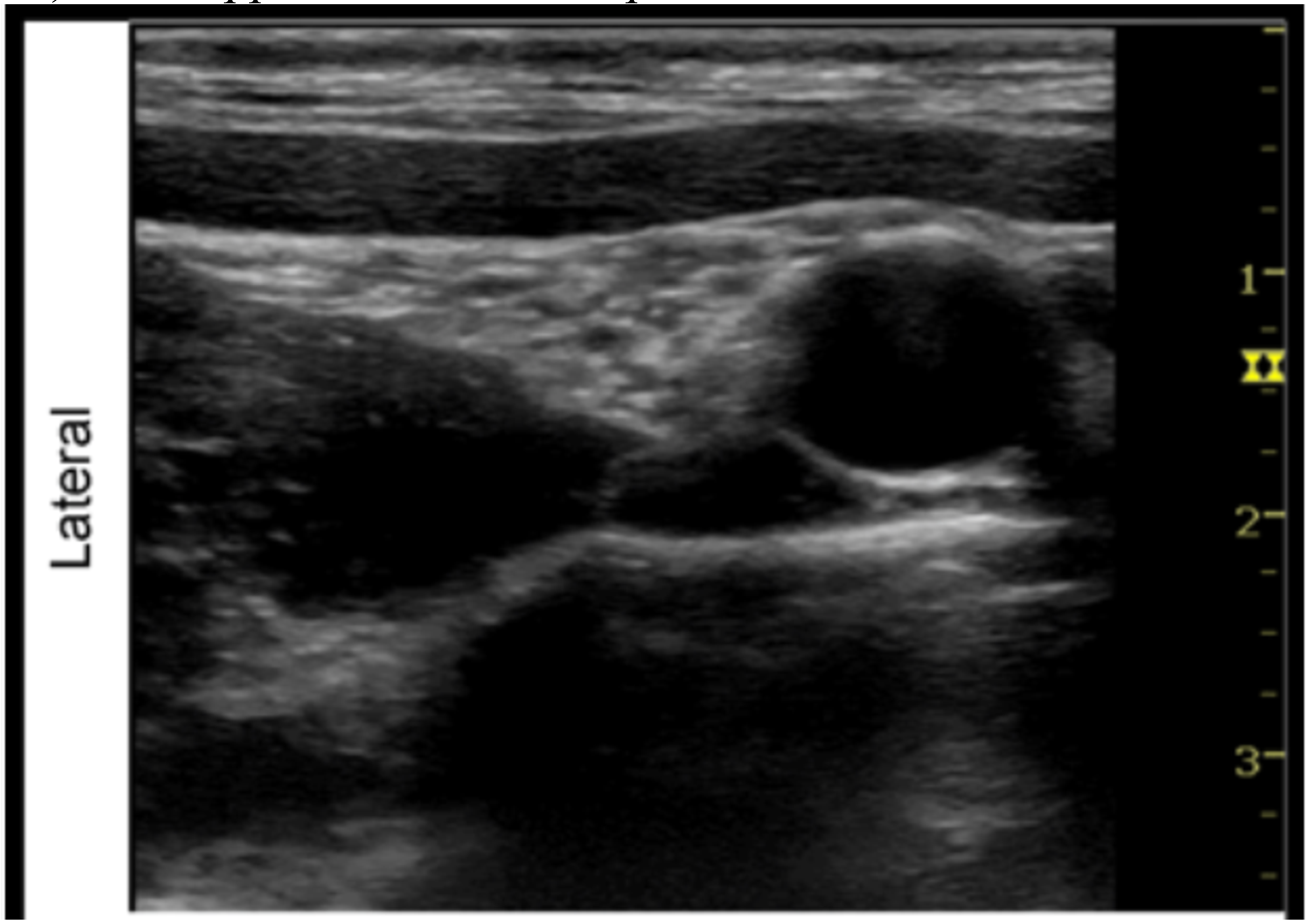


[Imagen consultada 1/28/24 desde

https://accessanesthesiology.mhmedical.com/data/books/hadzpnb2/m_hadzpnb2_c030f003.png]

A. Axilar

B. Infraclavicular

C. Supraclavicular

D. Interescalénico

16) Verdadero o falso: la mayoría de las lesiones nerviosas causadas por bloqueos de nervios periféricos se resuelven espontáneamente.

A, Verdadero

B. Falso

17) Un hombre de 70 kilogramos presenta para una reducción abierta y fijación interna para una fractura de tibia. Quiere realizar los bloqueos poplíteo y safeno. ¿Cuál es el volumen máximo de bupivacaína al 0,5% que se puede usar en este paciente? (Utilice la dosis máxima de bupivacaína de 2,5 mg/kg)

A. 25 mililitros

B. 35 mililitros

C. 45 mililitros

D. 55 mililitros

18) Además de las medidas habituales de soporte vital básico y avanzado, ¿qué tratamiento también debería incorporarse al tratamiento de la toxicidad sistémica del anestésico local?

A) Norepinefrina

B) Propofol

C) Vasopresina

D) Emulsión lipídica (Intralipid)

19) ¿Cuál es una ventaja del abordaje supraclavicular para el bloqueo del plexo braquial en comparación con el abordaje interescalénico?

A. La cara medial de la mano se anestesia de forma más fiable.

B. El riesgo de neumotórax es menor

C. El sitio es más compresible y flexible en caso de que se produzca sangrado.

D. La técnica es más fácil de realizar.

20) ¿Qué nervio no es una rama del nervio ciático?

A. Peroneo común

B. Tibia posterior

C. Sural

D. Safeno

21) ¿Qué parte del miembro inferior no puede anestesiarse mediante un bloqueo del nervio femoral?

A. El muslo medial

B. La pierna medial

C. El músculo cuádriceps

D. Piel que recubre la parte lateral del muslo.

22) ¿Qué parte del miembro inferior se anestesia mediante un bloqueo del nervio femoral?

A. Porción medial de la pierna

B. Planta del pie

C. Cara posterior del muslo

D. Tobillo y pie laterales

23) ¿Entre qué dos estructuras normalmente se pueden encontrar las raíces nerviosas en el abordaje interescalénico?

A) Músculos escalenos anterior y medio

B) Músculos escalenos posterior y medio

C) Músculos escalenos anterior y esternocleidomastoideo

D) Músculos escalenos medio y esternocleidomastoideo

24) ¿Cuál es la dosis en bolo inicial de emulsión lipídica que se debe administrar a un paciente para tratar la toxicidad del anestésico local?

A) 0,25 mL/kg

B) 1,5 mL/kg

C) 5 mL/kg

D) 7,5 mL/kg

**Key**

1. A
2. B
3. C
4. D
5. C
6. C
7. B
8. D
9. C
10. B
11. B
12. B
13. C
14. A
15. C
16. A
17. B
18. D
19. A
20. D
21. D
22. A
23. A
24. B

Reference:

1. Brouillette MA, Aidoo AJ, Hondras MA, Boateng NA, Antwi-Kusi A, Addison W, et al. Regional anesthesia training model for resource-limited settings: a prospective single-center observational study with pre-post evaluations. Reg Anesth Pain Med. 2020 Jul;45(7):528–35. PMID: 32447288. doi: 10.1136/rapm-2020-101550.

This is a Multimedia Appendix to a full manuscript published in the J Med Internet Res. For full copyright and citation information see http://dx.doi.org/10.2196/jmir.84181
