# Supplementary material for: Ultrasound-Guided Regional Anesthesia in a Resource-Limited Hospital: Prospective Pilot Study of a Hybrid Training Program
Source: JMIR Med Educ. 2026 Jan 8;12:e84181. doi: 10.2196/84181 (PMC12828311; doi:10.2196/84181)
Supplement: Multimedia Appendix 6 [file mededu_v12i1e84181_app6.docx]

**Global Rating Scale for Procedural Skills[1]**

| Preparation for procedure | 1  Did not organize equipment well. Has to stop procedure frequently to prepare equipment | 2 | 3  Equipment generally organized. Occasionally has to stop and prepare items | 4 | 5  All equipment neatly organized, prepared, and ready for use |
| --- | --- | --- | --- | --- | --- |
| Respect for tissue | 1  Frequently used unnecessary force on tissue or caused damage | 2 | 3  Careful handling of tissue but occasionally caused unintentional damage | 4 | 5  Consistently handled tissues appropriately with minimal damage |
| Time and motion | 1  Many unnecessary moves | 2 | 3  Efficient time/motion but some unnecessary moves | 4 | 5  Clear economy of movement and maximum efficiency |
| Instrument handling | 1  Repeatedly makes tentative or awkward moves with instruments | 2 | 3  Competent use of instruments but occasionally appeared stiff or awkward | 4 | 5  Fluid moves with instruments and no awkwardness |
| Flow of procedure | 1  Frequently stopped procedure and seemed unsure of next move | 2 | 3  Demonstrated some forward planning with reasonable progression of procedure | 4 | 5  Obviously planned course of procedure with effortless flow from one move to the next |
| Knowledge of procedure | 1  Deficient knowledge | 2 | 3  Knew all important steps of procedure | 4 | 5  Demonstrated familiarity with all aspects of procedure |
| Overall performance | 1  Very poor | 2 | 3  Competent | 4 | 5  Clearly superior |

Reference:

1. Chuan A, Graham PL, Wong DM, Barrington MJ, Auyong DB, Cameron AJ, et al. Design and validation of the Regional Anaesthesia Procedural Skills Assessment Tool. Anaesthesia. 2015 Dec;70(12):1401–11. PMID: 26558857. doi: 10.1111/anae.13266.

This is a Multimedia Appendix to a full manuscript published in the J Med Internet Res. For full copyright and citation information see http://dx.doi.org/10.2196/jmir.84181
