# Supplementary material for: Ultrasound-Guided Regional Anesthesia in a Resource-Limited Hospital: Prospective Pilot Study of a Hybrid Training Program
Source: JMIR Med Educ. 2026 Jan 8;12:e84181. doi: 10.2196/84181 (PMC12828311; doi:10.2196/84181)
Supplement: Multimedia Appendix 7 [file mededu_v12i1e84181_app7.pdf]

Fecha: ____________________ Médico Anestesiólogo: ______________________________

Cirugía: _________________________________

Complicaciones quirúrgicas: ☐ No ☐ Sí: ____________________________________

Tipo de anestesia: ☐ General ☐ Sedacíon ☐ Neuroaxial ☐ Bloqueo de nervio

**Información del paciente**	Edad: ________ Sexo: ☐ Hombre ☐ Mujer<br>Peso: __________ Altura: __________ Puntuación de ASA: __________<br>Comorbilidades: ☐ No ☐ Sí: ________________________________

**Bloque de Nervio**	☐ Interescalénico<br>☐ Supraclavicular<br>☐ Femoral<br>☐ Safeno (canal aductor)<br>☐ Ciático<br>☐ Poplíteo<br>☐ Otro: ________________	Lateralidad: ☐ Izquierda ☐ Derecha<br><br>Premedicación:<br>☐ No ☐ Sí: ______________________<br><br>Preparacíon:<br>☐ Consentimiento ☐ Preparación estéril<br>☐ Pausa ☐ Monitores

Anestésico Local: Tipo y concentracíon

☐ Bupivacaína: _________________%<br>☐ Lidocaína: ___________________%<br>☐ Otro	Volumen (mL):	Aditivos:<br>☐ No ☐ Sí: ___________

**Datos del procedimiento**	Números de intentos: __________<br>Parestesias: ☐ No ☐ Sí<br>Sangre aspirada: ☐ No ☐ Sí<br>Dolor al inyectar: ☐ No ☐ Sí<br>Complicaciones: ☐ No ☐ Sí: ______________________

Resultado de bloqueo

Alivio del dolor:<br>☐ No ☐ Sí	Bloqueo adecuado:<br>☐ Sí ☐ No - Intervención ____________________________________
