# Supplementary material for: Ultrasound-Guided Regional Anesthesia in a Resource-Limited Hospital: Prospective Pilot Study of a Hybrid Training Program
Source: JMIR Med Educ. 2026 Jan 8;12:e84181. doi: 10.2196/84181 (PMC12828311; doi:10.2196/84181)
Supplement: Multimedia Appendix 8 [file mededu_v12i1e84181_app8.docx]

**INFORMED CONSENT FOR PROCEDURES**

I declare and acknowledge that the anesthesiologist has explained to me the type of procedure planned, the risks and benefits, as well as the alternative options available. It has been explained to me that no guarantees or promises can be made regarding the results of the procedure. Although they are rare, unexpected complications can occur.

I understand that these services offered are part of a training program. Those involved in the procedure may be in training and require supervision. General information related to this procedure may be collected to evaluate the program. However, the data will be kept confidential and no personal information will be collected.

**Peripheral nerve block**

Technique: Medicine is injected near the nerves of the arm or leg. The medicine numbs part of your body.

Expected result: Temporary loss of sensitivity and the ability to move in part or all of the limb. Pain relief for a period of time in which pain is controlled.

Specific risks: Bruising or soreness, injury to blood vessels, bleeding, infection, permanent weakness, numbness, or pain from nerve injury, respiratory/cardiovascular collapse, ineffective blockade.

___________________________________ ____Left_________________Right_______

Block Type Location

I have read this form or someone has read this form to me. I understand what the form says. I have had the opportunity to ask questions and have them answered. I have enough information to give my permission.

______________________________ ______________________________

Patient Name Anesthesia Provider Name

______________________________ ______________________________

Patient Signature Anesthesia Provider Signature

______________________________ ______________________________

Relationship to patient (if other person) Date Time

______________________________

Date Time

**CONSENTIMIENTO INFORMADO PARA PROCEDIMIENTOS**

Declaro y reconozco que el profesional anestesiólogo me ha explicado el tipo de procedimiento planeado, los riesgos y beneficios, así como también las opciones disponibles. Me han explicado que no se pueden hacer garantías ni promesas con respecto a los resultados del procedimiento. A pesar de ser infrecuentes, pueden ocurrir complicaciones inesperadas.

Entiendo que estos servicios ofrecidos son parte de un programa de capacitación. Quienes participan en el procedimiento pueden estar en formación y requerir supervisión. La información general relacionada con este procedimiento puede recopilarse para evaluar el programa. Sin embargo, los hechos se mantendrán confidenciales y no se recopilará ninguna información personal.

**Bloqueo de nervio periférico**

Técnica: Se inyecta medicamento cerca de los nervios del brazo o pierna. El medicamento insensibiliza es parte de su cuerpo.

Resultado esperado: Pérdida temporal de sensibilidad y de la capacidad de moverse en parte o toda la extremidad. Alivio del dolor por un periodo de tiempo en que se controla del dolor.

Riesgos específicos: Moretones o sentirse adolorado/a, lesión a vasos sanguíneos, hemorragia, infección, debilidad permanente, insensibilidad, o dolor por lesión a los nervios, colapso respiratorio/cardiovascular, bloqueo inefectivo.

___________________________________ __ Izquierda________Derecha ____________

Tipo de bloqueo Lateralidad

He leído este formulario o alguien me ha leído este formulario. Comprendo lo que el formulario dice. He tenido oportunidad de hacer preguntas y de que me las respondan. Tengo suficiente información para dar mi permiso.

______________________________ ______________________________

Nombre del paciente Nombre del proveedor de anestesia

______________________________ ______________________________

Firma del paciente Firma del proveedor de anestesia

______________________________ ______________________________

Relación con el paciente (si otra persona) Fecha Hora

______________________________

Fecha Hora

This is a Multimedia Appendix to a full manuscript published in the J Med Internet Res. For full copyright and citation information see http://dx.doi.org/10.2196/jmir.84181
