# Supplementary material for: Ultrasound-Guided Regional Anesthesia in a Resource-Limited Hospital: Prospective Pilot Study of a Hybrid Training Program
Source: JMIR Med Educ. 2026 Jan 8;12:e84181. doi: 10.2196/84181 (PMC12828311; doi:10.2196/84181)
Supplement: Multimedia Appendix 9 [file mededu_v12i1e84181_app9.docx]

**Multimedia Appendix 9:** Baseline characteristics and pre-program survey results from anesthesiologists at the Hospital Nacional de Coatepeque.

| **Questions** | | **Value** |
| --- | --- | --- |
| **Participant demographics** | |  |
|  | Participants, n | 7 |
|  | Females, n (%) | 4 (57%) |
|  | Years of experience, mean (SD) | 9.1 (5.1) |
| **Have you used ultrasound previously? n (%)** | |  |
|  | Yes | 0 (0%) |
|  | No | 7 (100%) |
| **Do you have prior experience with blocks? n (%)** | |  |
|  | Yes | 7 (100%) |
|  | No | 0 (0%) |
| **What blocks have you performed?^a^ n (%)** | |  |
|  | Supraclavicular | 2 (29%) |
|  | Axillary | 5 (71%) |
|  | Peripheral nerve (radial, medial, ulnar) | 4 (57%) |
|  | Ankle | 5 (71%) |
|  | Sciatic | 2 (29%) |
|  | Scalp | 1 (14%) |
|  | Interscalene, infraclavicular, finger, femoral, saphenous, fascial plane | 0 (0%) |
| **Is regional anesthesia important for your practice? n (%)** | | |
|  | Yes | 6 (86%) |
|  | No | 1 (14%) |
| **What type of block would be most useful in your practice?^a^ n (%)** | | |
|  | Shoulder | 7 (100%) |
|  | Proximal arm | 6 (85%) |
|  | Distal arm | 4 (57%) |
|  | Leg | 4 (57%) |
|  | Thigh | 3 (43%) |
|  | Foot | 3 (43%) |
| **Were peripheral nerve blocks taught in residency? n (%)** | | |
|  | Yes | 5 (71%) |
|  | No | 2 (29%) |
| **Where have you learned about regional anesthesia?^a^ n (%)** | | |
|  | Online independent study | 3 (43%) |
|  | Online training program | 1 (14%) |
|  | Textbook | 2 (29%) |
|  | Conference | 1 (14%) |
| **What is your preferred learning style?^a^ n (%)** | |  |
|  | Workshops/clinical practice | 7 (100%) |
|  | Reading online resources | 2 (29%) |
|  | Reading textbooks | 2 (29%) |
|  | Conference | 1 (14%) |
| **What is your primary motivation for participating in this program?^a^ n (%)** | | |
|  | Learn new clinical skills | 6 (85%) |
|  | Patient outcomes | 5 (71%) |
|  | Work satisfaction | 2 (29%) |
|  | Patient satisfaction | 4 (57%) |
|  | Decreased resource utilization | 3 (43%) |
| **What are the biggest barriers to implementing regional anesthesia?^a^ n (%)** | | |
|  | Lack of supplies | 5 (71%) |
|  | Lack of personnel/time | 3 (43%) |
|  | Lack of training | 6 (85%) |

^a^Multiple responses allowed per participant.

This is a Multimedia Appendix to a full manuscript published in the J Med Internet Res. For full copyright and citation information see http://dx.doi.org/10.2196/jmir.84181
