# Supplementary material for: Ultrasound-Guided Regional Anesthesia in a Resource-Limited Hospital: Prospective Pilot Study of a Hybrid Training Program
Source: JMIR Med Educ. 2026 Jan 8;12:e84181. doi: 10.2196/84181 (PMC12828311; doi:10.2196/84181)
Supplement: Multimedia Appendix 10 [file mededu_v12i1e84181_app10.docx]

**Inter-rater reliability (IRR) for global rating scale assessment scores**

**Table 1:** Intraclass Correlation Coefficient by skill.

| **Skill** | **Estimate** | **95% CI** | **P-value** |
| --- | --- | --- | --- |
| 1 | 1 | (1.00, 1.00) | <0.001 |
| 2 | 0.37 | (0.00, 1.00) | 0.19 |
| 3 | 0.62 | (0.000002, 1.000000) | 0.049 |
| 4 | 0.73 | (0.000002, 1.000000) | 0.02 |
| 5^*^ | 0.000002 | (0.000002, 0.000069) | 0.5 |
| 6^*^ | 0.000002 | (0.000002, 0.000069) | 0.5 |
| Overall | 0.69 | (0.31, 0.88) | 0.03 |

**Table 2:** Weighted Kappa by skill.

| **Skill** | **Estimate** | **95% CI** | **P-value** |
| --- | --- | --- | --- |
| 1 | 1 | (1.0, 1.0) | 0.008 |
| 2 | 0.22 | (−0.40, 1.00) | 0.35 |
| 3 | 0.59 | (0.00, 1.00) | 0.088 |
| 4 | 0.7 | (0.00, 1.00) | 0.053 |
| 5^*^ | 0 | (0.0, 0.0) | 1 |
| 6^*^ | 0 | (0.0, 0.0) | 1 |
| Overall | 0.63 | (0.17, 0.86) | 0.058 |

^*^Skills 5 and 6 only differed by 1 point each across all participants. However, due to low variance in scores (nearly all patients scored 1 point in skill 5: “flow of procedure” and 2 points in skill 6: “knowledge of procedure”) unable to reliable compute IRR.

This is a Multimedia Appendix to a full manuscript published in the J Med Internet Res. For full copyright and citation information see http://dx.doi.org/10.2196/jmir.84181
