# Supplementary material for: Ultrasound-Guided Regional Anesthesia in a Resource-Limited Hospital: Prospective Pilot Study of a Hybrid Training Program
Source: JMIR Med Educ. 2026 Jan 8;12:e84181. doi: 10.2196/84181 (PMC12828311; doi:10.2196/84181)
Supplement: Multimedia Appendix 11 [file mededu_v12i1e84181_app11.docx]

**Multimedia Appendix 11**

**Table 1**: Characteristics of Patients and Procedures Performed During the UGRA Training Program.

| **Characteristic** | | **Value** |
| --- | --- | --- |
| Case demographics | |  |
|  | Patients, n | 147 |
|  | Peripheral nerve blocks, n | 187 |
| Patient information | | |
|  | Age (years), mean (SD) | 42.4 (20.8) |
|  | Females, number (%) | 43 (29%) |
|  | ASA score, mean (SD) | 1.4 (0.63) |
|  | Weight (kg), mean (SD) | 64.1 (18.6) |
| Block indications | | |
|  | Anesthetic, n (%) | 156 (83.4%) |
|  | Analgesic, n (%) | 31 (16.6%) |
| Block type | | |
|  | Interscalene, n (%) | 10 (6.8%) |
|  | Supraclavicular, n (%) | 66 (44.9%) |
|  | Femoral, n (%) | 35 (23.8%) |
|  | Adductor canal, n (%) | 25 (17.0%) |
|  | Popliteal, n (%) | 40 (27.2%) |
|  | Other, n (%) | 11 (7.5%) |
| Surgery type | | |
|  | Clavicle, n (%) | 8 (5.4%) |
|  | Shoulder/upper arm, n (%) | 9, (6.1%) |
|  | Lower arm/hand, n (%) | 64 (43.5%) |
|  | Upper leg, n (%) | 13 (8.8%) |
|  | Lower leg/foot, n (%) | 51 (34.7%) |
|  | Other, n (%) | 2 (1.4%) |

This is a Multimedia Appendix to a full manuscript published in the J Med Internet Res. For full copyright and citation information see http://dx.doi.org/10.2196/jmir.84181
